# Advances in NIR-II Fluorescent Nanoprobes: Design Principles, Optical Engineering, and Emerging Translational Directions

**DOI:** 10.3390/mi16121371

**Published:** 2025-12-01

**Authors:** Nargish Parvin, Mohammad Aslam, Md Najib Alam, Tapas K. Mandal

**Affiliations:** 1 School of Mechanical Engineering, Yeungnam University, Gyeongsan 38541, Republic of Korea; nargish.parvin@gmail.com; 2 School of Chemical Engineering, Yeungnam University, Gyeongsan 38541, Republic of Korea; mohammadaslam13@gmail.com

**Keywords:** fluorescent nanoprobes, biomedical imaging, disease diagnosis, quantum dots, in vivo biosensing

## Abstract

Fluorescent nanoprobes operating in the NIR-II window have gained considerable attention for biomedical imaging because of their deep-tissue penetration, reduced scattering, and high spatial resolution. Their tunable optical behavior, flexible surface chemistry, and capacity for multifunctional design enable sensitive detection and targeted visualization of biological structures in vivo. This review highlights recent advances in the design and optical engineering of four widely studied NIR-II nanoprobe families: quantum dots, carbon dots, upconversion nanoparticles, and dye-doped silica nanoparticles. These materials were selected because they offer well-defined architectures, controllable emission properties, and substantial mechanistic insight supporting discussions of imaging performance and translational potential. Particular focus is placed on emerging strategies for activatable, targeted, and ratiometric probe construction. Recent efforts addressing biosafety, large-scale synthesis, optical stability, and early preclinical validation are also summarized to clarify the current progress and remaining challenges that influence clinical readiness. By outlining these developments, this review provides an updated and focused perspective on how engineered NIR-II nanoprobes are advancing toward practical use in biomedical imaging and precision diagnostics.

## 1. Introduction

Biomedical imaging has become one of the most important tools in modern medicine. It provides clinicians with the ability to look inside the human body without surgical procedures and to detect abnormalities at very early stages of disease. Traditional imaging systems such as X-ray, computed tomography (CT), magnetic resonance imaging (MRI), positron emission tomography (PET), and ultrasound have transformed diagnosis and treatment planning. Each of these technologies has unique strengths. For example, MRI offers excellent soft tissue contrast, CT gives detailed structural information, and PET can monitor metabolic activity in tumors or organs [1]. Despite these advantages, they often fail to provide molecular-level information, which is essential for detecting diseases at the earliest stages. Early diagnosis is directly linked to improved patient survival. For instance, when cancers are detected before they metastasize, five-year survival rates can increase dramatically compared to detection at later stages [2]. Biomedical imaging helps achieve this goal by identifying subtle metabolic or structural changes that appear long before clinical symptoms. In addition, non-invasive imaging allows for repeated monitoring over time, which is critical for assessing how well therapies are working and for detecting possible recurrence. Imaging also plays a key role in guiding interventions. In surgical oncology, fluorescence-guided surgery enables surgeons to distinguish malignant from healthy tissue in real time, which reduces the risk of incomplete tumor removal and improves patient outcomes [3]. Similarly, imaging-guided biopsies allow more precise sampling, increasing diagnostic accuracy. Beyond clinical applications, imaging is essential in biomedical research. It helps reveal disease mechanisms, test drug delivery systems, and evaluate therapeutic responses in both preclinical models and human studies [4]. As imaging technologies continue to evolve, combining better resolution, higher sensitivity, and molecular specificity, their role in preventive medicine and personalized treatment will only expand.

Fluorescent nanoprobes are nanoscale materials engineered to emit light when excited by specific wavelengths. They provide unique opportunities for non-invasive imaging because of their tunable optical properties, high sensitivity, and potential for specific targeting. First, they can be engineered for high sensitivity and specificity. Fluorescent nanoprobes can detect small numbers of biomarkers at the molecular level, often before structural abnormalities are visible in conventional imaging. Functionalization with antibodies, peptides, or small molecules allows them to selectively bind to disease-associated targets such as overexpressed receptors in tumors [5]. Second, their optical properties can be precisely tuned. Quantum dots, carbon dots, upconversion nanoparticles, and dye-doped silica nanoparticles can all be designed to emit at specific wavelengths. In particular, probes that operate in the near-infrared (NIR) or second near-infrared window (NIR-II, 1000–1700 nm) are highly valuable, since tissue absorption and scattering are much lower in this region, enabling deeper penetration and higher resolution imaging [6]. Recent studies have demonstrated NIR-II nanoprobes achieving real-time visualization of vascular structures and tumor lesions several millimeters beneath the skin [7]. Among the available spectral windows, the NIR-II region has attracted particular interest due to its reduced scattering and improved deep-tissue visualization. A wide range of NIR-I and NIR-II fluorophores has been reported, including single-walled carbon nanotubes, aggregation-induced emission dots, and polymer-based emissive systems. Although these materials are important contributors to the field, the present review focuses on four representative nanoprobe families: quantum dots, carbon dots, upconversion nanoparticles, and dye-doped silica nanoparticles. These classes were selected because their structural architectures, optical tuning strategies, and biological behaviors are well characterized, allowing a mechanistic discussion of design principles and translational considerations. Including all materials would broaden the scope beyond the intended depth of analysis; therefore, relevant work on other fluorophore systems is acknowledged but not covered in detail.

**Figure 1:**
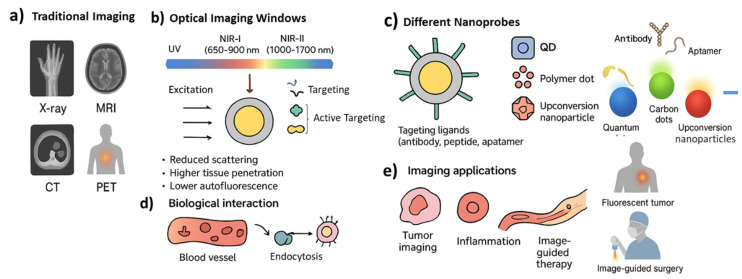
Schematic illustrating how nanoprobes improve non-invasive biomedical imaging. (**a**) Traditional imaging; (**b**) excitation emission windows and targeting pathways; (**c**) different nanoprobes; (**d**) biological interaction; (**e**) deep-tissue imaging advantages and combined diagnostic therapeutic functions, providing a clearer contrast with conventional imaging methods.

Third, fluorescent nanoprobes enable real-time and dynamic imaging. They make it possible to observe biological processes such as drug distribution, cellular uptake, or tumor microenvironment changes as they happen inside the body. This dynamic monitoring is particularly important for tracking therapeutic responses and adjusting treatments accordingly [8]. Finally, fluorescent nanoprobes can be multifunctional. They can combine diagnostic and therapeutic features in a single platform, a strategy often described as theranostics. For example, a nanoparticle may carry a fluorescent dye for imaging while also delivering an anticancer drug, or it may respond to internal signals such as pH or enzymatic activity to switch on its fluorescence only at disease sites [9,10,11,12]. This reduces background signals and increases accuracy. Despite their promise, some challenges remain. Issues such as long-term toxicity, accumulation of non-degradable particles, photobleaching, and limited clinical approval pathways must still be addressed [13,14]. However, progress is being made with biocompatible and biodegradable nanomaterials, as well as improved imaging systems capable of detecting very low probe concentrations. Figure 1 illustrates the paradigm shift from traditional macroscopic imaging techniques to nanoscale molecular imaging using fluorescent nanoprobes. While conventional methods such as MRI or PET rely on physical or metabolic contrasts, fluorescent nanoprobes exploit optical emission at specific wavelengths to provide molecular and functional information with subcellular precision. The figure also highlights how fluorescent nanoprobes improve biomedical imaging by combining optimized optical behavior with engineered biological interactions. The diagram highlights the benefits of using the NIR-I and NIR-II windows, where reduced scattering and minimal tissue autofluorescence allow deeper and clearer visualization than conventional fluorescence methods. The figure also outlines the modular design of nanoprobes, including core shell structures and surface ligands that promote selective targeting and controlled biodistribution. These design elements enable the probes to accumulate at disease sites through both passive and active mechanisms. The final panel illustrates key applications such as tumor detection, inflammation mapping, and image-guided therapy, emphasizing how nanoprobes support not only diagnostic imaging but also theranostic strategies in modern medicine.

### Scope and Objectives of This Review

• To summarize recent advances in fluorescent nanoprobes for biomedical imaging and disease diagnosis.
• To focus on four main classes of nanoprobes: quantum dots, carbon dots, upconversion nanoparticles, and dye-doped silica nanoparticles.
• To highlight functionalization strategies, including targeted, activatable, and ratiometric probes, especially those operating in the NIR and NIR-II regions.
• To discuss biomedical applications such as early disease detection, real-time monitoring, and image-guided surgery.
• To evaluate translational challenges including toxicity, reproducibility of synthesis, and regulatory approval.
• To provide a comparative overview of different fluorescent nanoprobe systems in terms of their optical and biological properties.
• To explore the design strategies that improve specificity, minimize background signals, and increase imaging depth.
• To review applications that demonstrate clinical relevance, with a focus on in vivo imaging and biosensing.
• To identify the barriers that hinder the translation of nanoprobes from laboratory to bedside.
• To propose future directions such as the integration of artificial intelligence, multimodal imaging, and precision medicine approaches.

Although a wide range of NIR-I and NIR-II fluorophores have been reported, including SWCNTs, AIE dots, and polymer-based emissive systems, the present review focuses on quantum dots, carbon dots, upconversion nanoparticles, and dye-doped silica nanoparticles. These four classes were selected because they provide well-defined structural architectures, tunable optical characteristics, and extensive preclinical datasets that allow a mechanistic discussion of design parameters and translational requirements. Other important NIR-II materials have been covered comprehensively in recent dedicated reviews, and including all available systems here would limit the depth of analysis. This review summarizes recent advances in NIR-II fluorescent nanoprobes with emphasis on their design principles, optical engineering, and emerging translational progress. By focusing on four widely studied nanoprobe families, the review highlights how structural control, emission tuning, and surface chemistry influence imaging performance in biological systems. Particular attention is given to targeted, activatable, and ratiometric probe designs, as well as ongoing efforts to improve biocompatibility, stability, and large-scale production. These aspects align with the revised scope defined in the title and aim to provide an updated, focused perspective on the evolving landscape of NIR-II imaging nanomaterials.

## 2. Fundamentals of Fluorescent Nanoprobes

### 2.1. Basic Principles of Fluorescence and Imaging

**Figure 2:**
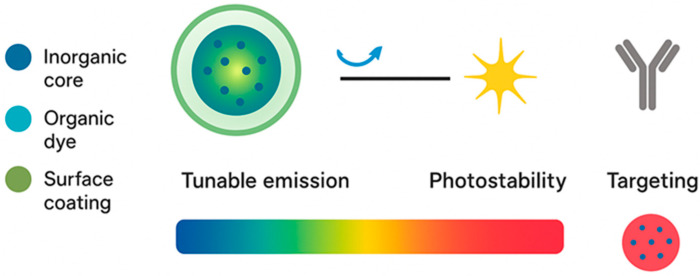
Fundamentals of fluorescent nanoprobes and their key functional characteristics. The schematic illustrates the structural composition and core principles governing the performance of fluorescent nanoprobes. These nanosystems typically comprise an inorganic or organic emissive core, an organic dye or dopant component, and a surface coating that enhances colloidal stability and enables bioconjugation. The emission wavelength can be finely tuned through changes in size, composition, and surface chemistry, offering control over the fluorescence spectrum from visible to near-infrared regions. Their high photostability ensures long-term imaging performance, while surface functionalization with biomolecules such as antibodies or peptides allows selective targeting of biological tissues or molecular markers.

Fluorescence is a photophysical process where a molecule or nanoparticle absorbs photons, transitions to an excited state, and then releases part of that energy as lower-energy photons. This emitted light has a longer wavelength than the excitation light, a property known as the Stokes shift. The two most important quantitative measures are the quantum yield, which describes how efficiently absorbed photons are converted into emitted photons, and the fluorescence lifetime, which is the average time a particle stays in the excited state before returning to the ground state [15]. These parameters define the strength, stability, and information content of the fluorescence signal. Biomedical imaging exploits these principles to provide real-time visualization of biological processes. For surface or shallow tissue imaging, wide-field fluorescence microscopy is commonly used because it is fast and simple. For greater depth and resolution, confocal and multiphoton microscopes reject out-of-focus light and enable three-dimensional reconstruction of biological structures [16]. In addition, fluorescence lifetime imaging microscopy (FLIM) extends beyond intensity measurements by separating signals based on lifetime, which reflects environmental factors such as pH, binding state, or molecular crowding. FLIM has become important in vivo because it can reduce background interference from tissue autofluorescence and allows quantitative comparisons independent of probe concentration [17,18]. A central challenge for fluorescence imaging in tissues is optical penetration. Visible light is strongly absorbed and scattered by hemoglobin, melanin, and water. Therefore, the use of near-infrared (NIR) light has gained attention. The NIR window, and particularly the second NIR region (1000–1700 nm), allows photons to penetrate deeper into tissues with less scattering and absorption. Fluorescent nanoprobes tuned to emit in this spectral region provide higher resolution and allow imaging several millimeters below the skin surface [19,20]. Fluorescent nanoprobes also introduce nanoscale control of photophysics. Unlike single dye molecules, nanoparticles can host many fluorophores in a protective matrix or can be intrinsically emissive themselves, as in the case of quantum dots or carbon dots. Encapsulation protects dyes from photobleaching and quenching, while the nanoscale environment alters emission spectra and lifetimes. Furthermore, nanoprobes can be engineered to respond to local biochemical conditions. Activatable probes remain dark until triggered by enzymatic activity or redox changes, producing high contrast only at disease sites [21]. These principles illustrate how fluorescence imaging has evolved from simple intensity detection into a sophisticated molecular tool for biomedical research and diagnosis. Figure 2 highlights the essential physicochemical and optical parameters that define the performance of fluorescent nanoprobes in biomedical imaging.

Optical characteristics such as tunable emission, quantum yield, and fluorescence lifetime are dictated by the core material and its electronic structure, while surface coatings and passivation layers minimize non-radiative decay and photobleaching. The diagram underscores how modifying nanoparticle size, doping concentration, or surface ligands enables emission tuning across the visible-to-NIR spectrum, which is crucial for deep-tissue imaging with reduced scattering and autofluorescence. Additionally, the surface functional layer serves multiple roles: it enhances colloidal stability in physiological environments, improves biocompatibility, and provides reactive sites for conjugation with targeting ligands. Together, these features translate into superior brightness, photostability, and targeting precision, distinguishing fluorescent nanoprobes from conventional small-molecule fluorophores. This integration of structural, optical, and functional design principles underpins their success in advanced diagnostic and therapeutic applications.

**Figure 3:**
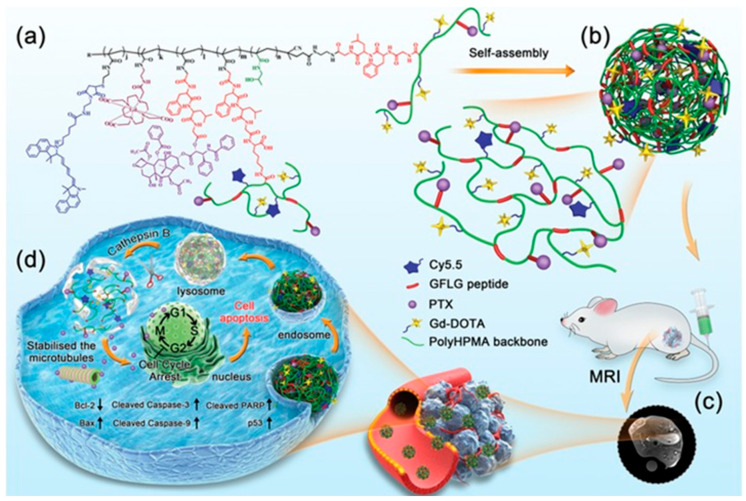
Schematic representation of cathepsin B-responsive biodegradable theranostic nanoparticles based on branched pHPMA conjugates. (**a**) Structural design of branched pHPMA-PTX-Gd conjugates. (**b**) Self-assembly of amphiphilic conjugates into BP-PTX-Gd nanoparticles. (**c**) In vivo MRI visualization of nanoparticle accumulation at tumor sites through the enhanced permeability and retention (EPR) effect. (**d**) Cellular uptake via lysosomal endocytosis, enzymatic degradation in cathepsin B-rich environments, and controlled paclitaxel release leading to microtubule stabilization and apoptosis induction. Reproduced with permission from ref. [21], Wiley-VCH Verlag GmbH & Co. CC BY 4.0.

A more detailed example can be seen in Figure 3, which illustrates the multifunctional design and mechanism of cathepsin B-responsive theranostic nanoparticles constructed from branched poly (2-hydroxypropyl methacrylamide) (pHPMA) conjugated with paclitaxel (PTX) and gadolinium (Gd) [22]. The structural concept leverages amphiphilic branches that self-assemble into nanoscale particles capable of both therapy and imaging. After intravenous administration, these nanoparticles exploit the enhanced permeability and retention (EPR) effect to preferentially accumulate at tumor sites, which can be monitored non-invasively via MRI owing to the Gd chelates. Once internalized by cancer cells through lysosomal pathways, the nanoparticles encounter elevated cathepsin B activity, leading to site-specific enzymatic degradation of the polymeric carrier. This process triggers the controlled release of PTX, which stabilizes microtubules and induces apoptosis selectively in the tumor microenvironment. The schematic highlights the synergy between targeted drug delivery and diagnostic imaging, offering a promising platform for precision oncology.

### 2.2. Key Optical and Physicochemical Properties

The effectiveness of any fluorescent nanoprobe depends on a balance between its optical and physicochemical characteristics. These optical parameters include absorption spectrum, emission wavelength, quantum yield, brightness, photostability, and fluorescence lifetime, while physicochemical properties include size, surface charge, colloidal stability, solubility, degradability, and bioconjugation capacity. This section focuses on material-dependent factors that specifically influence NIR-II probe performance rather than broad qualitative advantages. Brightness is determined by the absorption cross-section and quantum yield. A bright nanoprobe allows detection at very low doses, which is critical for reducing systemic toxicity [23]. Photostability is equally important, since many organic dyes bleach rapidly under continuous illumination. In contrast, inorganic probes such as quantum dots and upconversion nanoparticles show far superior resistance to bleaching [24,25]. Recent nanomaterial designs employ core-shell semiconductor structures, surface-passivated lanthanide hosts, and heteroatom-doped carbon matrices to enhance both brightness and photostability within the NIR-II window. The emission wavelength is central for in vivo imaging. Visible fluorophores are often limited by autofluorescence from tissues, while NIR and NIR-II emitting nanoprobes show higher penetration and reduced background [20]. For example, recent studies using NIR-II emitting nanoparticles have visualized vascular structures and tumor lesions with clarity not achievable using visible dyes. Fluorescence lifetime offers another dimension. It depends not only on the material but also on environmental conditions such as local oxygen concentration, pH, or binding interactions. Lifetime imaging reduces dependence on probe concentration and excitation power, giving more reliable quantitative data in heterogeneous tissues. Lifetime-sensitive nanoprobes are now being designed for tracking drug release or enzymatic activity directly inside living systems [17,26,27,28]. Lifetime tuning through defect-state manipulation, lanthanide-induced long-lived emission, and controlled energy-transfer pathways enables quantitative imaging independent of probe concentration. Physicochemical characteristics influence biodistribution and safety. Nanoparticles smaller than 6 nm may undergo renal clearance, while larger structures accumulate in the liver and spleen [29]. Surface charge affects protein-corona formation; neutral or zwitterionic coatings reduce nonspecific interactions, and PEGylation enhances circulation time [30].

Degradability is critical for safety. Persistent inorganic particles may accumulate in tissues, raising toxicity concerns. For this reason, carbon dots and biodegradable organic nanoprobes have attracted increasing attention as safer alternatives [31,32]. Surface chemistry must also support stable conjugation with targeting ligands without compromising colloidal stability.

### 2.3. Advantages over Conventional Probes

Fluorescent nanoprobes provide several clear advantages over conventional small-molecule fluorophores and other imaging agents. This section highlights design-driven and mechanistic improvements specific to modern nanoscale emitters. Nanoparticles can encapsulate or present multiple fluorophores, or they can exploit quantum confinement effects, giving them much higher brightness than a single dye molecule. This allows detection at low concentrations, which reduces toxicity risks [33]. Photostability: Unlike traditional dyes that bleach rapidly, quantum dots, upconversion nanoparticles, and certain metal nanoclusters are resistant to photobleaching. This stability is essential for long surgical procedures and longitudinal imaging studies [34]. Next-generation platforms such as nonblinking quantum dots, dopant-stabilized upconversion nanoparticles, and alloyed semiconductor nanocrystals provide improved resistance to intensity fluctuations and thermal quenching. Spectral tunability across visible, NIR, and NIR-II wavelengths enables multiplexed imaging of distinct biological targets [35]. Activatable and ratiometric fluorescent nanoprobes provide enhanced signal specificity by responding to biochemical cues such as pH, redox potential, and enzymatic activity [36]. Emerging activatable systems integrate tumor-microenvironment triggers, dual-band ratiometric outputs, and logic-gated responses to achieve high-contrast in vivo imaging. Multifunctionality allows nanoprobes to integrate imaging agents, therapeutic cargos, and targeting ligands on a single platform [37]. Advanced examples include NIR-II photothermal chemotherapy hybrids, ROS-responsive therapeutic nanoprobes, and immunomodulatory imaging constructs enabling real-time monitoring of therapeutic response. NIR-II probes further enable superior spatial resolution and deep-tissue imaging compared with visible fluorophores [38]. Table 1 summarizes essential optical and physicochemical parameters governing nanoprobe performance, highlighting how tunable emission, environmental responsiveness, and multifunctional integration differentiate fluorescent nanoprobes from conventional small-molecule fluorophores.

## 3. Classes of NIR-II Fluorescent Nanoprobes

The following sections focus on four representative nanoprobe families that offer well-characterized photophysical behavior and extensive evidence supporting their use in NIR-II biomedical imaging.

### 3.1. Quantum Dots

Quantum dots (QDs) are semiconductor nanocrystals typically ranging from 2 to 10 nm in size. Their most distinctive property is quantum confinement, where electronic and optical behaviors are strongly determined by particle size. As a result, QDs display size-tunable emission, allowing researchers to generate nearly any emission color by controlling nanocrystal diameter during synthesis [39,40]. The basic structure of QDs consists of an inorganic semiconductor core, often cadmium selenide (CdSe), cadmium telluride (CdTe), or indium phosphide (InP), coated with a shell such as zinc sulfide (ZnS) to improve stability and quantum yield. The core-shell design minimizes surface trap states, which would otherwise quench fluorescence, and provides higher brightness and resistance to photobleaching [41]. Synthesis approaches for QDs include colloidal chemical routes, hydrothermal methods, and microwave-assisted reactions. Colloidal synthesis is widely adopted because it enables precise control of particle size and surface passivation. Surface modification is also critical: ligands such as thiols, phosphines, and polymers are often used to stabilize QDs in aqueous environments. Further functionalization with biomolecules (antibodies, peptides, aptamers) allows targeting specific receptors on diseased cells or tissues [42,43,44]. A unique feature of QDs is their broad absorption spectra and narrow emission peaks. This makes them highly suitable for multiplex imaging, where several QD populations with different emission colors can be excited simultaneously with a single light source. Their photostability surpasses that of organic fluorophores, enabling long-term imaging experiments without significant loss of signal. Additionally, QDs often exhibit higher quantum yields compared to dyes, which enhances sensitivity for detecting low-abundance biomarkers [45]. However, despite these advantages, toxicity concerns especially with heavy metal-based QDs remain a barrier for clinical translation. Cadmium leakage from degraded QDs can generate reactive oxygen species and damage cellular structures. To address this, research has shifted toward developing cadmium-free QDs, such as InP/ZnS and carbon-based alternatives, that maintain favorable optical properties while minimizing risks.

QDs have shown broad applications in in vitro and in vivo disease imaging. In cancer research, QDs conjugated with tumor-targeting ligands enable visualization of tumor margins and micrometastases. For example, QDs functionalized with anti-HER2 antibodies have been used to selectively label HER2-positive breast cancer cells, allowing precise imaging of tumor heterogeneity [46]. QDs are also valuable in angiography and vascular imaging, where their long circulation times and tunable emission allow high-resolution visualization of blood vessels [47]. Their narrow emission spectra make them suitable for multiplexed imaging, enabling simultaneous monitoring of tumor vasculature and immune cell infiltration. In neurological imaging, QDs have been applied to track neuronal activity and protein transport along axons. For instance, QDs conjugated with synaptic vesicle proteins allow researchers to observe synaptic dynamics in living neurons, which is crucial for understanding neurodegenerative disorders such as Alzheimer’s disease [48]. Another promising application is intraoperative guidance. QDs emitting in the near-infrared region have been tested for real-time imaging during surgery, providing surgeons with enhanced visualization of tumor boundaries and sentinel lymph nodes. This improves resection accuracy and reduces recurrence rates [49]. Finally, QDs have been investigated in infectious disease diagnostics, where QD-based immunoassays show superior sensitivity compared to conventional fluorescent dyes. QD-conjugated antibodies can detect viral proteins at very low concentrations, which is critical for early disease diagnosis [50]. Overall, QDs represent one of the most versatile classes of fluorescent nanoprobes. Their tunable emission, brightness, and photostability make them powerful tools for biological imaging, though their safety profiles must be carefully evaluated before widespread clinical adoption. Recent years have seen progress toward low-toxicity, clinically relevant quantum dots and expanded activity in the NIR-II band. Work has focused on cadmium-free cores (for example InP-based systems) and on surface chemistries that reduce ion leaching while preserving brightness and narrow emission. These developments have improved biocompatibility and enabled high-contrast vascular and tumor imaging in the NIR window, moving QDs closer to translational studies and multifunctional nanocomposites for guided therapy.

### 3.2. Carbon Dots

Carbon dots (CDs) are considered one of the most promising fluorescent nanoprobes due to their remarkable stability, low toxicity, and easily adjustable emission properties. Unlike traditional organic dyes, which tend to degrade under prolonged illumination, CDs demonstrate outstanding photostability, enabling their use in long-term imaging and repeated excitation cycles [11,51]. The high resistance to photobleaching originates from the strong carbon–carbon bonds in their graphitic core, as well as the presence of surface functional groups that stabilize emissive states. This stability makes them particularly suitable for continuous monitoring of dynamic biological processes, where conventional dyes fail to provide reliable signals [43]. Equally important is the biocompatibility of CDs. Many semiconductor quantum dots raise safety concerns due to heavy metal content, whereas CDs are composed primarily of carbon, oxygen, and hydrogen, with optional heteroatoms such as nitrogen or sulfur. Several in vitro and in vivo studies have confirmed their low cytotoxicity and minimal immunogenic response, especially when prepared from natural precursors like citric acid, amino acids, or biomass waste [52]. In addition, their small size, usually below 10 nm, facilitates renal clearance and reduces the likelihood of long-term accumulation in vital organs, an advantage over larger inorganic nanoprobes [53,54]. Another unique feature of CDs is their tunability. Their fluorescence can be adjusted across the visible to near-infrared spectrum by manipulating synthetic parameters, surface chemistry, and heteroatom doping. For instance, nitrogen-doped CDs often exhibit enhanced quantum yields and red-shifted emissions compared to undoped CDs [55]. Similarly, sulfur or phosphorus doping modifies electronic structures, enabling better charge transfer and stronger fluorescence. The emission tunability extends to excitation-dependent fluorescence, where the emission color shifts with the excitation wavelength, a property rarely seen in conventional dyes [56,57]. This flexibility allows CDs to be engineered for specific imaging windows, including the NIR region, which is optimal for deep-tissue imaging with reduced background interference. The tunability also extends to lifetime properties, enabling the development of CDs with distinct fluorescence lifetimes suitable for multiplex imaging and lifetime-based biosensing. Moreover, CDs exhibit environment-sensitive fluorescence, meaning their emission can respond to pH, metal ions, or redox states, further expanding their role in diagnostic applications [58]. Overall, the combination of high photostability, excellent biocompatibility, and versatile tunability makes CDs an exceptional class of nanoprobes that can overcome many limitations of conventional fluorophores and heavy-metal-based nanocrystals.

The biomedical applications of CDs are diverse, ranging from fundamental bioimaging to advanced theranostics. Their optical stability and safety profile have made them increasingly attractive for both in vitro and in vivo uses. One of the most prominent areas is tumor imaging. Targeted CDs functionalized with ligands such as folic acid, transferrin, or specific peptides can selectively accumulate in tumor tissues overexpressing the corresponding receptors. For example, folic acid-modified CDs have successfully visualized folate receptor-positive breast cancer cells with high selectivity, producing strong fluorescence contrast even in complex biological environments [59]. Such probes not only assist in early cancer detection but also help in monitoring tumor progression and therapy response. CDs have also been integrated into biosensing platforms. Their fluorescence is highly sensitive to local biochemical changes, enabling the detection of ions (Cu^2+^, Fe^3+^, Hg^2+^), small biomolecules (glucose, dopamine), and enzymes (alkaline phosphatase, caspases). Ratiometric and turn-on/off fluorescence probes based on CDs provide improved accuracy over single-emission probes, as they minimize background interference and concentration-dependent variations [60]. In one study, CDs were designed to detect hydrogen peroxide, a reactive oxygen species closely linked with oxidative stress in cancer and cardiovascular diseases, demonstrating excellent sensitivity and selectivity [61]. In neuroimaging, CDs offer special advantages due to their ability to cross the blood–brain barrier (BBB). Several studies have reported successful delivery of CDs to brain tissues, enabling visualization of neuronal structures and monitoring neurotransmitter levels. For instance, dopamine-sensitive CDs have been used to track neurotransmitter release, opening possibilities for early diagnosis of Parkinson’s disease and other neurological disorders [62,63]. CDs are also being developed for in vivo imaging in the near-infrared (NIR) region, which provides deeper tissue penetration. By tuning surface chemistry and doping, researchers have produced NIR-emitting CDs that enable vascular imaging and real-time monitoring of tumor angiogenesis in small animal models [49]. These advances position CDs as safer alternatives to traditional NIR dyes like indocyanine green, which suffer from rapid clearance and photobleaching. Beyond diagnostics, CDs are now integrated into theranostic systems that combine imaging with therapy. CDs can serve as drug carriers, owing to their surface functional groups that allow conjugation with chemotherapeutic agents. For example, doxorubicin-loaded CDs have been used to deliver the drug selectively to tumor cells while simultaneously providing fluorescence for real-time tracking of drug distribution [64,65]. Similarly, CDs have been engineered as photothermal and photodynamic therapy agents, converting light into heat or reactive oxygen species to kill cancer cells while enabling simultaneous imaging [66]. Another expanding application is in regenerative medicine. CDs have been incorporated into scaffolds and hydrogels for tissue engineering, where they provide both imaging capabilities and bioactive cues that promote cell growth and differentiation [27,67,68]. Collectively, these biomedical applications highlight the broad potential of CDs. Their excellent safety profile, customizable optical features, and multifunctionality make them highly suitable for translational research. Future developments are expected to focus on large-scale, reproducible synthesis, deeper tissue imaging, and regulatory validation for clinical trials. Recent research has broadened the optical performance of carbon dots far beyond the visible spectrum. Controlled heteroatom doping, surface-state engineering, and molecular fusion strategies have enabled strong NIR-I and NIR-II emission, which significantly improves deep-tissue imaging. Several studies have also demonstrated long fluorescence lifetimes, stimuli-responsive switching, and tunable afterglow emission that support high-contrast tracking in complex biological environments. These advances have positioned carbon dots as versatile probes for multimodal imaging, metabolic sensing, and image-guided therapeutic interventions.

### 3.3. Upconversion Nanoparticles

#### 3.3.1. Mechanism of Upconversion Luminescence

Upconversion nanoparticles (UCNPs) are a unique class of fluorescent probes that convert low-energy near-infrared (NIR) photons into higher-energy visible or ultraviolet photons through a process known as upconversion luminescence (UCL). This process is fundamentally different from conventional fluorescence, where the emitted light has a longer wavelength than the excitation light. UCNPs instead exhibit anti-Stokes emission, meaning the emitted photons are of higher energy than the absorbed ones [69,70]. The mechanism of upconversion relies on lanthanide ions (such as Er^3+^, Tm^3+^, Yb^3+^) doped into an inorganic crystalline host matrix, commonly NaYF_4_, due to its low phonon energy and ability to minimize nonradiative losses [71]. The upconversion process typically involves multiple sequential photon absorption events mediated by the metastable energy states of the doped lanthanide ions. Among the major mechanisms are:

• **Excited-state absorption (ESA):** A single ion absorbs two or more photons in succession, moving stepwise to higher excited states before emitting a photon of shorter wavelength.
• **Energy transfer upconversion (ETU):** An energy donor ion (commonly Yb^3+^) absorbs NIR light and transfers the excitation energy to a nearby acceptor ion (such as Er^3+^ or Tm^3+^), which then emits higher-energy photons.
• **Photon avalanche (PA):** A positive feedback process where initial excitation triggers a chain of absorption and emission events, leading to rapid signal amplification.

Upconversion nanoparticles have undergone important redesign in recent years to enhance brightness and biological performance. Core–shell–shell structures, energy-migration layers, and dopant optimization have led to higher upconversion efficiency under low-power excitation. At the same time, surface modifications such as ligand-exchange engineering and polymer encapsulation now support targeted delivery, reduced aggregation, and improved stability in physiological media. These improvements have made UCNPs more suitable for real-time tracking, deep-tissue optical imaging, and activatable systems that respond to pH, enzymes, or redox cues. UCNPs show sharp emission peaks and long luminescence lifetimes due to the electronic transitions of lanthanide ions, which are shielded from environmental quenching by filled 5s and 5p orbitals. This leads to stable, highly reproducible signals, making them reliable for imaging and biosensing [72]. Recent studies have also advanced core shell UCNP architectures. Coating a doped core with an inert shell (e.g., NaYF_4_:Yb^3+^, Er^3+^@NaYF_4_) suppresses surface quenching and enhances quantum yield [73]. Multifunctional core shell designs can integrate multiple dopant ions or combine UCNPs with magnetic, plasmonic, or drug-loaded components for theranostic applications.

#### 3.3.2. Advantages in Deep-Tissue Imaging

UCNPs offer several advantages over conventional fluorescent nanoprobes, especially for deep-tissue imaging. First, UCNPs are typically excited in the NIR window (700–1000 nm), where biological tissues show minimal scattering and absorption. This allows deeper photon penetration and reduces background autofluorescence, which is a major limitation for visible fluorophores [18]. The second NIR window (1000–1700 nm) provides even greater penetration depths, and UCNPs designed for dual-NIR excitation and emission show excellent promise for in vivo imaging at depths of several millimeters [74]. Second, the anti-Stokes nature of UCNP emission ensures that autofluorescence, which usually arises from endogenous biomolecules excited by visible light, does not overlap with the upconverted signal. This leads to exceptionally high signal-to-background ratios in live tissue imaging [75]. Third, UCNPs exhibit outstanding photostability compared to organic dyes and quantum dots. Their emission intensity remains stable under prolonged excitation, making them ideal for long-term monitoring of biological events such as tumor progression, cell trafficking, or drug delivery [76]. UCNPs have been successfully applied in cancer imaging, where antibody- or peptide-functionalized UCNPs selectively bind to tumor biomarkers, enabling sensitive detection of small tumors or metastatic foci [,^77^]. In addition, UCNPs are used in intraoperative navigation, guiding surgeons during tumor resection by providing real-time fluorescence signals under NIR excitation. Another notable advantage is their multifunctionality. UCNPs can be co-loaded with therapeutic molecules or conjugated with photodynamic therapy (PDT) agents. Under NIR excitation, the upconverted emission activates photosensitizers to produce cytotoxic reactive oxygen species, enabling simultaneous imaging and therapy [78,79,80]. Finally, UCNPs are being explored for multiplexed imaging, as different lanthanide dopants produce distinct emission colors under the same excitation wavelength. This allows simultaneous monitoring of multiple biological targets, a key step toward precision diagnostics [81]. Taken together, the unique mechanism and superior imaging performance of UCNPs make them an invaluable tool for biomedical research and translational medicine.

### 3.4. Dye-Doped Silica Nanoparticles

Dye-doped silica nanoparticles (DDSNs) represent another important class of fluorescent nanoprobes. These materials consist of organic fluorescent dyes encapsulated within a silica matrix. The silica framework provides mechanical stability, chemical protection, and high biocompatibility, addressing the major weaknesses of free dyes such as photobleaching and poor aqueous solubility [82]. The design of DDSNs typically involves sol-gel synthesis or microemulsion techniques, where dye molecules are incorporated during nanoparticle formation. By covalently binding dyes to the silica network or entrapping them within mesoporous structures, leaching is minimized and fluorescence stability is maintained [83]. A major design consideration is the prevention of dye aggregation, which can lead to self-quenching. Silica encapsulation provides spatial separation between dye molecules, preserving fluorescence intensity and quantum yield. Furthermore, silica shells can be engineered with controlled porosity, thickness, and surface functionalization to tailor DDSNs for specific biomedical uses [84]. Another advantage of silica is its surface chemistry. Recent developments in dye-doped silica nanoparticles have focused on controlling dye packing, pore structure, and energy transfer pathways to achieve higher quantum yields and improved stability. Advances in mesoporous frameworks allow precise spatial confinement of dye molecules, reducing quenching and enhancing brightness. Hybrid dye–silica systems have also been tailored for afterglow luminescence, ratiometric sensing, and dual-modality imaging. In addition, redesigned PEGylation strategies and ligand-controlled surface modifications have improved in vivo compatibility and targeted accumulation, strengthening their potential for cancer diagnosis, biosensing, and theranostic platforms.

**Figure 4:**
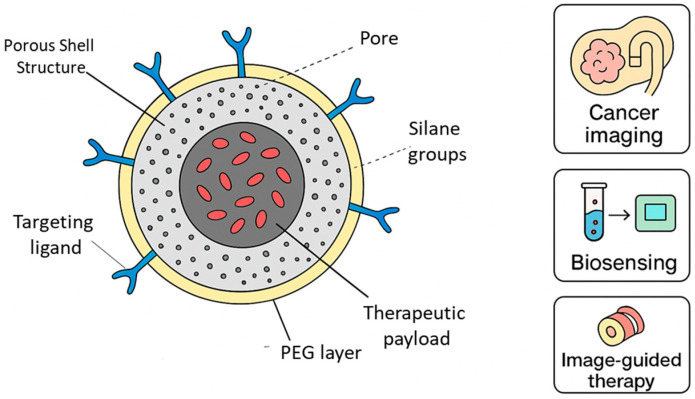
Structural layout and multifunctionality of dye-doped silica nanoparticles. The enhanced schematic includes the porous silica network, embedded dye molecules, PEG coating, ligand-binding domains, and optional drug-loading compartments. Representative biomedical applications such as cancer imaging, biosensing, and image-guided therapy are illustrated.

Functional groups on the nanoparticle surface allow for easy functionalization with biomolecules such as antibodies, peptides, and aptamers, enabling targeted imaging. Polyethylene glycol (PEG) or ionic coatings improve colloidal stability and extend circulation time in vivo [85]. DDSNs also provide multicolor and multimodal capabilities. By doping multiple dyes into the same particle, ratiometric or multiplexed imaging becomes possible. DDSNs can also be integrated with magnetic or plasmonic materials, producing hybrid nanostructures with both imaging and therapeutic potential [86]. Overall, the silica matrix not only protects encapsulated dyes but also ensures long-term stability and reproducibility, making DDSNs attractive for clinical translation. Figure 4 illustrates how dye-doped silica nanoparticles integrate structural and functional features to support a wide range of biomedical uses. The porous silica network provides a stable environment for encapsulating fluorescent dyes, which improves brightness and reduces photobleaching compared with free dyes in solution. The PEG surface layer enhances colloidal stability and minimizes nonspecific interactions during circulation, while the presence of silane groups and targeting ligands allows controlled functionalization for selective recognition of biological markers. The figure also includes an optional therapeutic region, highlighting how these particles can be adapted for combined imaging and drug delivery. Together, these design elements explain why dye-doped silica nanoparticles are widely used in cancer imaging, biosensing, and image-guided therapeutic applications.

**Figure 5:**
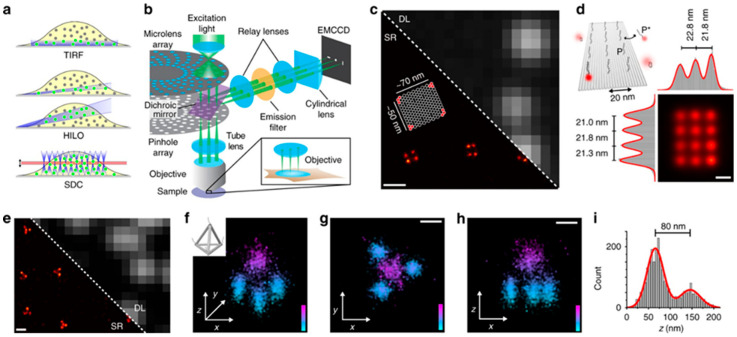
High-resolution DNA-PAINT imaging using spinning disk confocal (SDC) microscopy. (**a**) Comparison of illumination modes: TIRF for surface imaging, HILO for deeper optical sectioning, and SDC for focal-plane detection (red). (**b**) Diagram of the SDC system employing a cylindrical lens to achieve 3D super-localization through optical astigmatism. (**c**) DNA origami structures visualized by DNA-PAINT under diffraction-limited (DL) and super-resolution (SR) modes. (**d**) SDC–DNA-PAINT achieves sub-20 nm precision, matching the designed DNA origami geometry. (**e**–**i**) Two- and three-dimensional reconstructions of DNA origami tetrahedra, showing accurate structural alignment with designed dimensions (edge length ≈ 100 nm; height ≈ 80 nm). Scale bars: 200 nm (**c**,**e**), 50 nm (**g**,**h**), and 20 nm (**d**). Reproduced with permission from ref. [87], CC BY 4.0.

To evaluate fluorescent nanoprobes with high spatial precision, advanced optical imaging systems are often used alongside conventional fluorescence microscopy. Figure 5 is included to illustrate how high-resolution imaging modalities support the study of fluorescent nanoprobes. Techniques such as spinning disk confocal microscopy combined with DNA-PAINT allow researchers to map nanoprobe distribution, quantify binding events, and validate targeting accuracy at the nanoscale [87]. These approaches help correlate probe design with biological performance, especially when investigating molecular interactions or structural organization in cells and tissues. By providing this level of spatial detail, such imaging systems complement the nanoprobe platforms discussed in this review and aid in evaluating their diagnostic potential.

The versatility of DDSNs has led to a wide range of applications in diagnostics, biosensing, and molecular imaging. In cancer diagnostics, DDSNs functionalized with tumor-targeting ligands have been used to visualize tumor margins and detect circulating tumor cells. Their high fluorescence intensity allows identification of rare cancer cells in complex biological fluids with high sensitivity [88]. DDSNs are also widely applied in biosensing. For instance, pH-sensitive dyes encapsulated in silica nanoparticles have been used to monitor intracellular pH fluctuations, which are critical in cancer metabolism and apoptosis. Similarly, DDSNs doped with ion-sensitive dyes can detect calcium or potassium concentrations in living cells, enabling real-time monitoring of cellular signaling. In pathogen detection, DDSNs conjugated with antibodies against bacterial or viral antigens enable highly sensitive assays. Their photostability ensures consistent signal output, which is crucial for point-of-care diagnostic devices [89]. DDSNs also play a role in image-guided drug delivery. Mesoporous silica nanoparticles loaded with chemotherapeutic agents can be co-doped with dyes to allow simultaneous drug release tracking and tumor imaging. This dual functionality enhances therapeutic monitoring and reduces systemic toxicity [90,91,92]. Finally, DDSNs have been investigated for multiplexed biomarker detection. By incorporating multiple fluorescent dyes into a single nanoparticle, researchers can simultaneously track several disease-related analytes, a capability essential for early and accurate diagnosis of complex diseases like cancer and neurodegeneration [93]. Taken together, dye-doped silica nanoparticles combine the brightness and tunability of organic dyes with the stability and versatility of silica, making them a robust and adaptable class of nanoprobes for biomedical imaging and diagnostics. Here, Table 2 summarizes the principal classes of fluorescent nanoprobes: Quantum Dots, Carbon Dots, Upconversion Nanoparticles, and Dye-Doped Silica Nanoparticles. The table emphasizes their structural composition, photophysical mechanisms, and biomedical relevance. Each nanoprobe class exhibits distinct advantages: Quantum Dots provide high brightness and tunable emission; Carbon Dots offer superior biocompatibility and environmental responsiveness; Upconversion Nanoparticles enable deep-tissue imaging with minimal autofluorescence; and Dye-Doped Silica Nanoparticles ensure long-term stability and functional versatility. Together, these nanoprobes represent the foundation of modern fluorescence-based biomedical diagnostics and theranostics.

## 4. Functionalization and Targeting Strategies

### 4.1. Surface Modification and Bioconjugation

Surface functionalization is a central step in the design of fluorescent nanoprobes, as it directly influences their stability, solubility, circulation time, and biological interactions. The bare surface of many nanoparticles is prone to aggregation or nonspecific binding to biomolecules, which can reduce imaging precision and increase off-target effects. To overcome this, researchers employ surface modification strategies such as polymer coatings, ligand attachment, and covalent or noncovalent functionalization [94]. Polyethylene glycol (PEG) is among the most widely used coatings. PEGylation shields nanoparticles from recognition by the mononuclear phagocyte system, prolonging their circulation time in vivo. PEG chains also provide steric hindrance, reducing aggregation and enhancing colloidal stability in biological fluids [95]. Beyond PEG, zwitterionic polymers and polysaccharides such as dextran and chitosan have also been employed, offering not only stabilization but also additional functional groups for conjugation [96]. Bioconjugation allows nanoparticles to interact specifically with target biomolecules or receptors. This can be achieved by covalently attaching ligands such as antibodies, peptides, aptamers, or small molecules. For example, carbodiimide chemistry is commonly used to link carboxyl groups on nanoparticle surfaces with amine groups on biomolecules, while click chemistry provides a highly efficient and bio-orthogonal route for ligand attachment [97]. Noncovalent strategies, such as electrostatic interactions, hydrophobic interactions, or affinity-based approaches (biotin–streptavidin), also provide versatile routes for functionalization when reversible binding is desirable. Surface engineering additionally plays a role in minimizing cytotoxicity. For instance, silica or lipid coatings around quantum dots or upconversion nanoparticles provide a biocompatible barrier that prevents leaching of toxic ions [98]. Similarly, carbon dots and dye-doped silica nanoparticles can be tailored with amino, hydroxyl, or carboxyl groups to improve solubility and reduce protein adsorption in vivo. Overall, surface modification and bioconjugation strategies not only improve nanoparticle pharmacokinetics but also provide a molecular interface for targeting, biosensing, and therapeutic delivery. Their rational design is key to developing safe and effective fluorescent nanoprobes.

### 4.2. Tumor-Targeted and Organ-Specific Probes

**Figure 6:**
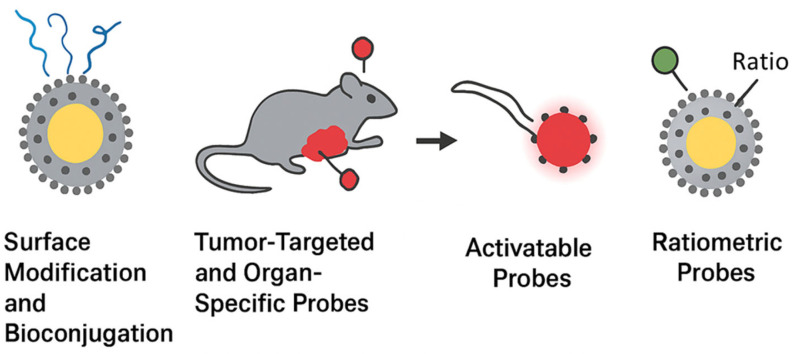
Overview of functionalization and targeting strategies in fluorescent nanoprobes. The schematic illustrates key approaches used to enhance nanoparticle performance and specificity in biomedical imaging, including surface modification and bioconjugation, tumor-targeted and organ-specific delivery, and advanced probe designs such as activatable and ratiometric systems.

One of the most transformative advances in nanomedicine is the development of tumor-targeted and organ-specific fluorescent probes, which provide selective accumulation in diseased tissues and minimize background interference. Targeting strategies can be divided into passive and active approaches. Passive targeting primarily relies on the enhanced permeability and retention (EPR) effect, where nanoparticles accumulate in tumors due to leaky vasculature and poor lymphatic drainage. However, the EPR effect alone is often insufficient for clinical translation because of variability between tumor types and patients [99]. To overcome this, active targeting has been developed, which involves functionalizing nanoparticle surfaces with ligands that bind to tumor-associated biomarkers. Ligands used for tumor targeting include antibodies (trastuzumab for HER2-positive breast cancer), peptides (RGD peptides for integrin αvβ3), aptamers (AS1411 targeting nucleolin), and small molecules (folic acid for folate receptors) [100]. These functionalized nanoprobes demonstrate enhanced tumor accumulation and uptake by cancer cells, thereby increasing imaging contrast. For example, folic acid decorated carbon dots have shown selective accumulation in ovarian cancer models, while antibody-modified quantum dots have successfully visualized HER2 expression in breast tumors. Beyond tumors, organ-specific targeting is gaining attention. Nanoprobes can be engineered to selectively accumulate in the liver, kidneys, lungs, or brain by exploiting tissue-specific transport mechanisms. For instance, glycyrrhetinic acid-modified nanoparticles show high affinity for hepatocytes, making them suitable for liver cancer imaging [101]. Similarly, small peptides such as angiopep-2 have been used to facilitate blood-brain barrier penetration, enabling fluorescent imaging of neurological diseases. Another promising area is immune cell-targeted probes, which allow tracking of macrophages, T cells, and dendritic cells during immunotherapy. Functionalization with specific antibodies or receptor ligands enables visualization of immune cell migration and infiltration into tumors [102]. Together, tumor-targeted and organ-specific nanoprobes hold significant promise for precision diagnostics. By improving specificity, they reduce systemic toxicity, enhance imaging resolution, and open possibilities for personalized treatment strategies. Figure 6 summarizes the principal strategies employed to functionalize fluorescent nanoprobes for precision imaging and diagnostics. Surface modification and bioconjugation are fundamental for improving nanoparticle colloidal stability, circulation time, and biocompatibility while providing reactive sites for ligand attachment. Tumor-targeted and organ-specific nanoprobes further enhance diagnostic accuracy through either passive accumulation via the enhanced permeability and retention (EPR) effect or active recognition of biomarkers using antibodies, peptides, or small molecules. Activatable probes introduce a new level of control by emitting fluorescence only in response to specific biological stimuli, thereby minimizing background noise and improving sensitivity. In contrast, ratiometric probes enable quantitative imaging through self-calibrating dual-emission signals that correct for environmental or instrumental variations. Collectively, these strategies represent an integrated design philosophy where surface chemistry, targeting, and signal modulation converge to produce high-performance nanoprobes capable of real-time, selective, and quantitative imaging in complex biological environments.

### 4.3. Activatable and Ratiometric Probes

Traditional fluorescent probes often suffer from constant emission regardless of their biological environment, which can lead to high background signals and poor sensitivity. To address this limitation, researchers have developed activatable probes that emit fluorescence only in response to specific stimuli, and ratiometric probes that provide quantitative information by measuring emission ratios. Activatable probes are engineered to respond to biochemical or physical triggers present in the tumor microenvironment or diseased tissues. These triggers include low pH, high glutathione concentration, elevated enzyme activity (e.g., matrix metalloproteinases, caspases), or hypoxic conditions. For example, upconversion nanoparticles functionalized with pH-sensitive polymers exhibit “off-on” luminescence in acidic tumor environments, improving contrast over non-activated probes [103]. Similarly, enzyme-cleavable peptide linkers have been incorporated into carbon dots and silica nanoparticles, which release strong fluorescence only after enzymatic cleavage, allowing precise detection of cancer-associated proteases [104]. Ratiometric probes provide a more quantitative approach to imaging by comparing two fluorescence signals: one that responds to the stimulus and one that remains constant as a reference. This self-calibration mechanism eliminates fluctuations due to probe concentration, photobleaching, or uneven illumination [105]. For instance, dual-emission carbon dots have been developed for ratiometric pH sensing in live cells, while dye-doped silica nanoparticles with dual fluorophores can simultaneously report intracellular ion concentrations and serve as an internal reference. In addition, ratiometric upconversion probes have been widely studied for oxygen sensing and redox monitoring in tissues, providing insights into tumor metabolism and hypoxia [106]. The ability to quantify biological parameters with high spatial and temporal resolution makes ratiometric probes highly valuable for precision diagnostics. The integration of activatable and ratiometric designs represents a major advancement in fluorescent nanoprobe technology. These smart systems not only enhance sensitivity and specificity but also provide real-time functional information, which is critical for early disease detection and therapeutic monitoring. Table 3 summarizes the major functionalization and targeting strategies employed in fluorescent nanoprobes for biomedical applications. Surface modification and bioconjugation approaches improve physicochemical stability and provide molecular recognition capabilities, while tumor- and organ-targeted nanoprobes enhance selective accumulation and imaging precision. Smart activatable and ratiometric designs further advance sensitivity and quantification in complex biological environments, representing key innovations toward clinically translatable nanodiagnostics.

## 5. Biomedical Applications of Fluorescent Nanoprobes

### 5.1. Early Disease Detection and Diagnosis

Early detection of diseases, particularly cancer, cardiovascular disorders, and infectious diseases, is critical for improving patient outcomes. Fluorescent nanoprobes have emerged as highly sensitive tools for this purpose because of their bright emission, tunable optical properties, and ability to selectively target biomarkers. By conjugating nanoparticles with ligands such as antibodies, peptides, or aptamers, disease-specific biomolecules can be recognized even at low concentrations, enabling preclinical and clinical detection at early stages [107]. Quantum dots, for example, have been functionalized with antibodies against tumor-associated antigens, allowing the identification of circulating tumor cells in blood samples. Their high photostability and brightness improve detection sensitivity compared to conventional dyes, enabling the capture of rare cells that might otherwise be missed [108]. Similarly, carbon dots conjugated with folic acid or transferrin can selectively accumulate in cancer cells expressing the corresponding receptors, producing strong fluorescence contrast and facilitating early tumor detection [109]. Fluorescent nanoprobes are also applied in infectious disease diagnostics. Pathogen-specific probes allow rapid visualization of bacteria or viruses in complex biological fluids. Upconversion nanoparticles have been integrated into immunoassays to detect viral antigens at sub-picomolar levels due to their high signal-to-background ratio and resistance to autofluorescence, significantly improving diagnostic accuracy [110]. Instead of relying on generic sensitivity improvements, current systems leverage anti-Stokes emission, narrowband lanthanide transitions, and high-affinity bioconjugation chemistries to enhance diagnostic precision.

Fluorescent nanoprobes can detect metabolic or biochemical changes associated with disease. Ratiometric carbon dots, for example, have been used to monitor intracellular pH, redox states, and reactive oxygen species, which often change during cancer progression or inflammation. Modern activatable probes incorporate enzyme-cleavable peptides, redox-triggered fluorogenic switches, and microenvironment-responsive linkers that generate a signal only in pathological tissues, improving diagnostic specificity. Such mechanisms suppress background fluorescence and enhance contrast between healthy and diseased regions [93]. Through these mechanism-focused designs, such as activatable, ratiometric, and receptor-targeted constructs, fluorescent nanoprobes provide precise, early-stage diagnostic signals suitable for non-invasive screening, point-of-care testing, and longitudinal disease monitoring.

### 5.2. Image-Guided Surgery

**Figure 7:**
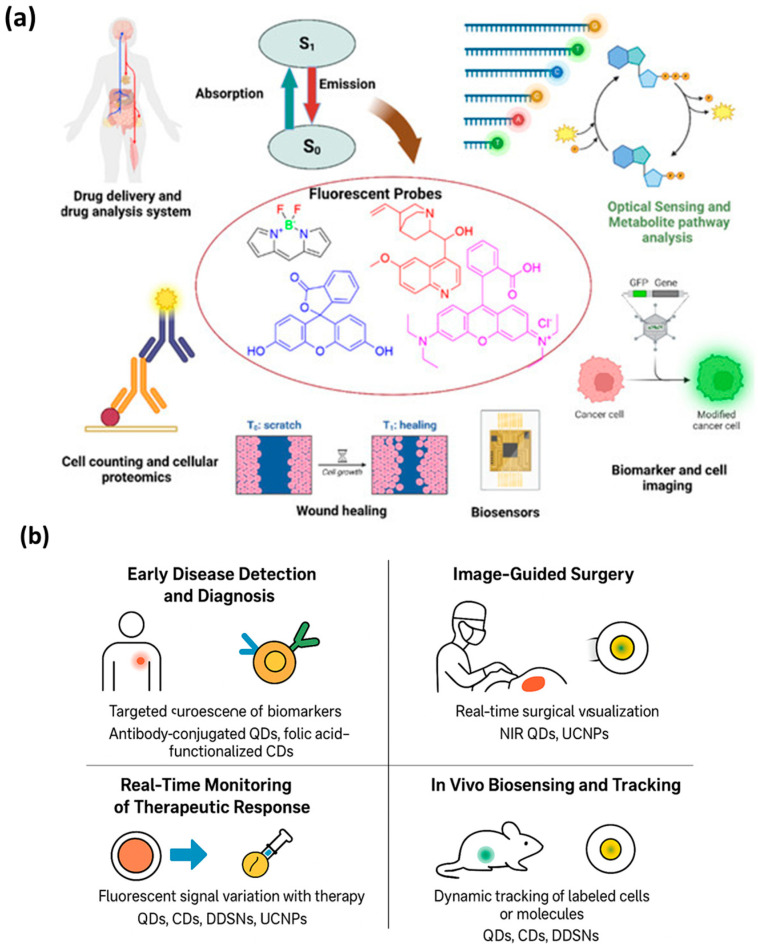
(**a**) Overview of biomedical uses of fluorescent probes. Fluorescence generated during the transition from the excited singlet state (S1) to the ground state (S0) enables diverse applications, including targeted drug delivery, cell analysis, wound repair assessment, biosensing, and molecular or cellular imaging. Abbreviations: S1—excited singlet state; S0—ground singlet state; GFP—green fluorescent protein. Adopted ref. [113], CC by 4.0. (**b**) Biomedical applications of fluorescent nanoprobes across diagnostic and therapeutic domains. The figure illustrates four major applications: (i) early disease detection and diagnosis through targeted fluorescence of disease biomarkers, (ii) image-guided surgery for real-time visualization of tumor margins, (iii) real-time monitoring of therapeutic response via fluorescence variation with treatment efficacy, and (iv) in vivo biosensing and tracking for dynamic observation of cells and biomolecules within living systems.

Surgical outcomes, particularly in oncology, depend heavily on accurately identifying tumor margins and metastatic tissues. Fluorescent nanoprobes have transformed this field by providing real-time intraoperative visualization. By emitting strong, stable fluorescence under NIR or visible light, these probes allow surgeons to distinguish between healthy and diseased tissue, minimizing incomplete resections and reducing recurrence rates [111]. Near-infrared-emitting quantum dots and upconversion nanoparticles are particularly suitable for deep-tissue imaging during surgery. NIR light penetrates several millimeters into tissue, enabling visualization of tumors beneath the surface, while reducing autofluorescence and phototoxicity [49]. Carbon dots and dye-doped silica nanoparticles have also been explored for image-guided surgery because of their biocompatibility, ease of surface functionalization, and high photostability [112]. Targeted nanoprobes enhance surgical precision by binding specifically to tumor-associated antigens. For example, antibodies or peptides conjugated to fluorescent nanoparticles accumulate in tumor margins, providing a sharp contrast that guides the resection process. Activatable probes add an extra layer of safety: fluorescence is only turned on in response to tumor microenvironment triggers, such as acidic pH or enzyme activity, further improving delineation of cancerous tissue. Recent studies demonstrate the effectiveness of nanoparticle-assisted image-guided surgery in multiple cancers, including breast, liver, and brain tumors. By enabling surgeons to visualize even micrometastatic lesions, fluorescent nanoprobes contribute to higher survival rates and better postoperative outcomes. Figure 7a illustrates the fundamental fluorescence mechanism emission from the excited singlet state (S1) to the ground state (S0)-and its translation into diverse biomedical functionalities. This photophysical process enables high-sensitivity detection, making fluorescent probes essential for applications such as imaging cellular structures, tracking biomolecules, and monitoring biological responses. Their versatility supports advanced techniques in drug delivery assessment, wound-healing evaluation, biosensing, and proteomic analysis, demonstrating how fluorescence underpins both diagnostic and analytical innovations in biomedicine [113]. And Figure 7b summarizes the diverse biomedical applications of fluorescent nanoprobes, demonstrating their versatility in bridging diagnostics and therapy. Through selective targeting and high fluorescence sensitivity, these nanoprobes enable early detection of diseases such as cancer and infections by identifying specific biomarkers at trace levels. Their application in image-guided surgery provides surgeons with real-time optical feedback, allowing precise tumor resection while minimizing damage to healthy tissues. In theranostics, fluorescent nanoprobes serve dual roles, acting both as drug carriers and imaging agents to monitor therapeutic responses dynamically. Furthermore, their integration into in vivo biosensing platforms enables real-time tracking of biological processes, immune cell movement, and molecular signaling pathways in living organisms. Together, these applications highlight the crucial role of fluorescent nanoprobes in advancing precision medicine, offering non-invasive, high-resolution tools for diagnosis, treatment monitoring, and biomedical research.

### 5.3. Real-Time Monitoring of Therapeutic Response

Monitoring how tissues respond to therapy is essential for precision medicine, allowing clinicians to adjust treatment plans in real-time. Fluorescent nanoprobes provide a non-invasive means to track therapeutic efficacy at the cellular and molecular level. For chemotherapy and targeted therapy, nanoparticles can be loaded with drugs and simultaneously serve as imaging agents. Quantum dots, carbon dots, and dye-doped silica nanoparticles have been developed as theranostic platforms, enabling real-time tracking of drug delivery, release, and accumulation in tumors [114]. Changes in fluorescence intensity or lifetime can indicate the extent of drug uptake, cell apoptosis, or tumor regression. Fluorescent nanoprobes are also useful for monitoring photodynamic and photothermal therapies. Upconversion nanoparticles, for example, can activate photosensitizers under NIR light while simultaneously providing emission for imaging. By observing fluorescence changes during treatment, clinicians can assess whether sufficient reactive oxygen species have been generated to induce tumor cell death [115]. Activatable probes further enhance real-time monitoring. Probes that respond to enzymatic activity, redox state, or pH provide functional information about tissue status. For instance, ratiometric carbon dots can indicate intracellular oxidative stress levels during chemotherapy, offering immediate feedback on treatment effectiveness [116]. The integration of fluorescent nanoprobes into treatment monitoring allows personalized therapy, reduces unnecessary exposure to ineffective drugs, and provides quantitative data for clinical decision-making.

### 5.4. In Vivo Biosensing and Tracking

In vivo biosensing is a critical application of fluorescent nanoprobes, allowing dynamic observation of cell migration, biomolecule distribution, and metabolic processes in live organisms. Fluorescent nanoparticles can be engineered to target specific cells or organelles and report on their behavior in real-time. For instance, immune cells labeled with quantum dots or carbon dots can be tracked during immunotherapy, providing insights into cell trafficking, infiltration into tumors, and interaction with the tumor microenvironment [117]. Similarly, nanoparticles can monitor the biodistribution of therapeutics, identify off-target accumulation, and assess pharmacokinetics without the need for invasive sampling. Fluorescent nanoprobes also serve as biosensors for metabolites and ions. Ratiometric carbon dots or dye-doped silica nanoparticles respond to pH, glucose, calcium, or reactive oxygen species levels, providing spatial and temporal maps of biochemical changes in living organisms [118]. Upconversion nanoparticles are particularly effective in this role because their NIR excitation allows deep-tissue imaging with minimal background interference [49]. Additionally, in vivo tracking using fluorescent nanoprobes facilitates longitudinal studies, enabling researchers and clinicians to observe disease progression, immune responses, or therapeutic outcomes over time. This capability is invaluable in both preclinical research and future clinical translation. By combining high sensitivity, real-time reporting, and multifunctionality, fluorescent nanoprobes are poised to become indispensable tools in modern biomedical research and precision medicine. Here the Table 4 summarizes the diverse biomedical applications of fluorescent nanoprobes, encompassing early disease detection, image-guided surgery, therapeutic monitoring, and in vivo biosensing. Through precise surface engineering and optical optimization, these nanoprobes achieve exceptional sensitivity, photostability, and selectivity in biological environments. Their integration into clinical and research settings enables real-time visualization of disease progression, accurate tumor margin delineation, and functional monitoring of therapy responses, marking a transformative step toward personalized and precision medicine.

## 6. Translation from Bench to Bedside

The transition of fluorescent nanoprobes from laboratory research to clinical use involves a multistage development process. Preclinical studies focus on physicochemical characterization, in vitro efficacy, and in vivo biodistribution. Characterization ensures reproducible size, shape, surface charge, and fluorescence properties, which directly influence nanoparticle behavior in biological systems [119]. In vitro studies assess cellular uptake, cytotoxicity, targeting efficiency, and stability in biological media. For instance, quantum dots and carbon dots are tested against relevant cancer cell lines to determine specificity and potential off-target effects [120]. In vivo preclinical models, including small rodents and larger animals, provide critical data on biodistribution, clearance, pharmacokinetics, and preliminary efficacy. Functionalized nanoparticles are often labeled with NIR fluorophores or radiolabels to monitor tissue accumulation and clearance rates in real time. These studies reveal whether nanoparticles achieve sufficient tumor accumulation via passive (EPR effect) or active targeting, and whether undesired organ deposition occurs in the liver, spleen, or kidneys [121]. Once preclinical benchmarks are met, translational research advances toward clinical feasibility studies. These studies are designed to evaluate safety, dosing, and imaging efficacy in humans. Early-phase trials (Phase I/II) often focus on healthy volunteers or small patient cohorts, assessing tolerability, biodistribution, and preliminary diagnostic capability. For example, silica-coated quantum dots and dye-doped nanoparticles have undergone pilot imaging trials for sentinel lymph node detection, providing valuable proof-of-concept data.

Toxicity remains a major barrier to clinical translation. Nanoparticle composition, size, surface chemistry, and dosage directly influence biosafety. Semiconductor quantum dots containing cadmium pose significant cytotoxic risks, including oxidative stress, mitochondrial damage, and inflammation [122]. To mitigate this, researchers have developed cadmium-free alternatives such as indium phosphide or silicon-based quantum dots, which retain desirable optical properties while reducing heavy metal exposure [41]. Carbon dots and dye-doped silica nanoparticles exhibit lower toxicity due to their biocompatible composition, but long-term accumulation and immunogenicity remain concerns. Surface modifications, such as PEGylation or lipid coatings, reduce protein adsorption and immune recognition, improving systemic tolerability [123]. Biosafety studies also evaluate renal and hepatic clearance, as nanoparticles with prolonged retention can induce organ-specific toxicity. Smaller nanoparticles (<10 nm) tend to undergo renal clearance, while larger particles are often sequestered in the liver or spleen. Additionally, in vivo immunogenicity, complement activation, and hemocompatibility tests are essential to ensure that nanoparticles do not trigger adverse immune responses.

Regulatory approval for fluorescent nanoprobes is complex, as they straddle the boundary between diagnostics, imaging agents, and therapeutic devices. The FDA, EMA, and other regulatory bodies require rigorous documentation of manufacturing reproducibility, quality control, safety, and efficacy. Unlike traditional small-molecule drugs, nanoprobes require additional characterization for size distribution, surface chemistry, stability, and batch-to-batch consistency [124]. Clinical translation is further complicated by the diversity of nanomaterials. Each nanoparticle class, whether quantum dots, carbon dots, or upconversion nanoparticles, may present unique toxicological profiles and pharmacokinetics, necessitating tailored regulatory strategies. Furthermore, multifunctional nanoparticles that combine imaging and therapy are classified as combination products, requiring evaluation under both drug and device regulations, which increases development timelines and costs [125]. A common regulatory challenge is long-term safety evaluation, particularly for inorganic nanoparticles that may persist in organs. Standardized guidelines for preclinical testing and toxicological assessment are still evolving, making early consultation with regulatory agencies essential for successful translation.

Despite challenges, several fluorescent nanoprobes have entered clinical trials, primarily in cancer diagnostics and intraoperative imaging. For example, indocyanine green (ICG)-loaded silica nanoparticles have been tested for sentinel lymph node mapping in breast and gastric cancer, demonstrating improved visualization and resection accuracy compared with conventional dyes [126]. Another example involves cadmium-free quantum dots functionalized with tumor-targeting ligands, which have been tested in pilot imaging studies for gastrointestinal and breast cancers. These trials report high signal-to-background ratios, excellent photostability, and minimal adverse events, supporting feasibility for human use [127]. Upconversion nanoparticles are also under investigation for deep-tissue imaging applications, with early-phase studies exploring liver, pancreatic, and brain tumor imaging. These studies leverage the low tissue absorption of NIR light and the high photostability of UCNPs to visualize tumors in vivo without significant background interference [49]. Carbon dots, with their favorable safety profile and tunable fluorescence, are entering biosensing and diagnostic trials, particularly for detecting biomarkers in blood or urine samples. These trials evaluate detection sensitivity, specificity, and potential for early disease screening [128]. Overall, clinical translation of fluorescent nanoprobes is progressing steadily, with ongoing studies refining safety, targeting efficiency, and imaging accuracy. Future advancements are likely to integrate multimodal imaging, artificial intelligence–assisted image analysis, and multifunctional theranostic platforms to achieve precision diagnostics and personalized medicine.

Table 5 distills the multi-step pathway of translating fluorescent nanoprobes from laboratory concept to clinical reality, highlighting the critical hurdles and mitigation strategies at each stage. Initially, rigorous physicochemical and biological testing ensures reproducible performance and biocompatibility, which is foundational before any in vivo investigation. In living systems, achieving desirable pharmacokinetics and efficient tumor targeting with minimal off-target accumulation is essential, especially when moving toward human studies. Toxicity assessment remains perhaps the most formidable barrier: even trace leaching of heavy metal ions or chronic nanoprobe retention can raise regulatory alarms. Compounding this, the classification of fluorescent nanoprobes as imaging agents, devices, or drug-device hybrids demands rigorous quality control and consistency in manufacturing. Early-phase clinical trials must validate safety, biodistribution, and diagnostic efficacy, ideally outperforming or complementing existing modalities. Encouragingly, several nanoprobe constructs such as silica-encapsulated dyes and cadmium-free quantum dots have already entered pilot clinical testing, particularly for sentinel lymph node mapping. However, the future of translation likely lies in simplifying nanoprobe designs, integrating with other imaging modalities, leveraging smart (activatable) probes, and adopting AI-driven image analysis frameworks. Continued alignment between probe developers, clinicians, and regulatory agencies is essential to close the bench-to-bedside gap and bring advanced fluorescent nanoprobes into routine clinical use.

## 7. Challenges and Limitations

### 7.1. Biocompatibility and Long-Term Safety

Despite the remarkable potential of fluorescent nanoprobes, biocompatibility and long-term safety remain critical challenges that limit their clinical translation. The interaction of nanoparticles with biological systems is complex and influenced by factors such as size, shape, surface chemistry, and dosage. For instance, semiconductor quantum dots may release heavy metal ions like cadmium or lead under physiological conditions, causing oxidative stress, mitochondrial dysfunction, and cytotoxicity [129]. Even cadmium-free alternatives, such as indium phosphide or silicon-based quantum dots, require careful evaluation for potential immunogenicity or organ accumulation [130]. Carbon dots, dye-doped silica nanoparticles, and upconversion nanoparticles generally exhibit lower acute toxicity, but their long-term biodistribution and clearance are still areas of concern. Nanoparticles with poor biodegradability may accumulate in the liver, spleen, or kidneys, potentially causing chronic inflammation or organ dysfunction [131]. Additionally, repeated exposure may trigger immune responses, including complement activation or antibody production, which could alter pharmacokinetics and reduce probe efficacy. Surface coatings, such as polyethylene glycol (PEG), zwitterionic polymers, or lipid shells, can mitigate some of these risks by improving circulation time, reducing protein adsorption, and minimizing immune recognition. However, these modifications do not completely eliminate long-term retention issues, highlighting the need for systematic in vivo studies and standardization of biocompatibility testing for clinical translation [132]. Regulatory bodies, such as the FDA and EMA, now emphasize comprehensive toxicological assessment, including genotoxicity, reproductive toxicity, and chronic exposure studies, to ensure the safety of nanoprobes before approval. Addressing these concerns is essential to gain clinical acceptance and prevent adverse events that could compromise patient safety.

### 7.2. Stability and Reproducibility of Synthesis

**Figure 8:**
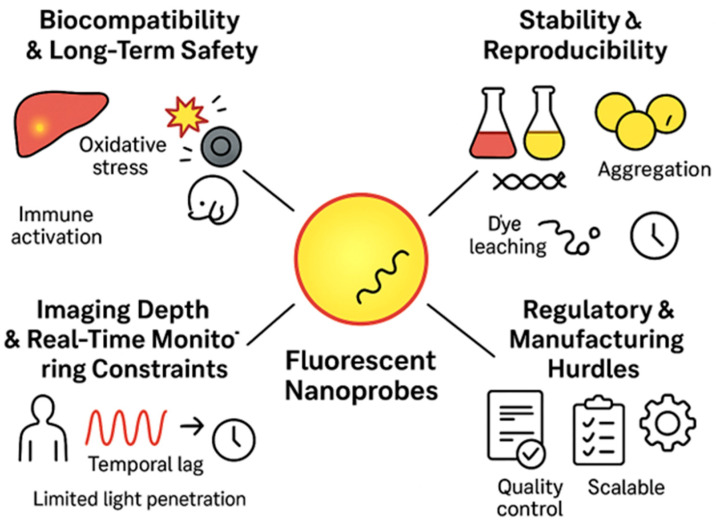
Key Challenges and Limitations in the Clinical Translation of Fluorescent Nanoprobes.

Another significant limitation is the stability and reproducibility of nanoparticle synthesis. Fluorescent nanoprobes must exhibit consistent optical properties, size distribution, and surface characteristics to ensure reliable imaging results. Variations in synthesis methods, precursor purity, reaction temperature, and dopant concentration can lead to batch-to-batch variability, affecting fluorescence intensity, emission wavelength, and biodistribution [133]. For instance, quantum dots and upconversion nanoparticles require precise control over crystal growth and dopant incorporation to achieve uniform emission profiles. Minor deviations during synthesis can result in heterogeneous populations, which compromise targeting specificity and quantification in biomedical applications [134]. Similarly, carbon dots and dye-doped silica nanoparticles can suffer from aggregation, dye leaching, or surface defect formation if synthesis conditions are not rigorously controlled. Long-term colloidal stability in biological media is also a challenge. Nanoparticles may aggregate in the presence of salts, proteins, or serum components, reducing bioavailability and altering biodistribution. Surface modification and functionalization strategies, while helpful, must be reproducible and scalable for clinical production [135]. Scalable manufacturing methods that maintain high reproducibility, stability, and batch consistency are necessary for regulatory approval and commercialization. Addressing these technical challenges requires collaboration between chemists, materials scientists, and biomedical engineers to develop standardized synthesis protocols. Figure 8 provides a comprehensive overview of the multifaceted challenges that hinder the clinical translation of fluorescent nanoprobes. Biocompatibility and long-term safety remain at the forefront, as nanoparticle accumulation, oxidative stress, and immune activation can compromise patient safety and therapeutic reliability. Stability and reproducibility issues further complicate translational progress, since minor variations in synthesis parameters or surface chemistry can alter fluorescence behavior, biodistribution, and biological performance. Moreover, imaging depth and real-time monitoring are constrained by tissue scattering, autofluorescence, and limited penetration of visible and near-infrared light, restricting the use of these probes in deep-tissue imaging. Beyond the scientific and technical barriers, regulatory and manufacturing challenges such as maintaining batch-to-batch consistency, achieving GMP compliance, and meeting toxicity evaluation standards pose additional obstacles to clinical adoption. Collectively, these challenges underscore the need for integrated interdisciplinary strategies combining materials science, biomedical engineering, and regulatory innovation to achieve safe and effective fluorescent nanoprobes for real-world biomedical applications.

### 7.3. Imaging Depth and Real-Time Monitoring Constraints

While fluorescent nanoprobes provide high sensitivity and specificity, imaging depth and real-time monitoring present inherent limitations, particularly for in vivo applications. Conventional visible-light-emitting probes are limited by tissue scattering and absorption, restricting effective imaging depth to a few millimeters [136]. Near-infrared (NIR) and upconversion nanoparticles partially overcome this limitation, allowing imaging several centimeters deep. However, signal attenuation, light scattering, and tissue autofluorescence still impose constraints, particularly in dense or highly vascularized tissues [137]. Real-time monitoring adds another layer of complexity. Rapid physiological processes, dynamic tissue movements, and nanoparticle clearance can lead to fluctuating signal intensity. Activatable and ratiometric probes improve signal-to-background ratio, but precise temporal resolution is limited by the kinetics of nanoparticle accumulation, activation, and emission [106]. Additionally, integrating imaging systems into clinical workflows remains challenging. High-sensitivity detectors, specialized excitation sources, and real-time image processing are often required for deep-tissue or rapid imaging applications. These technical requirements increase cost, complexity, and accessibility, particularly in resource-limited settings [138]. Addressing these limitations requires multimodal imaging strategies, combining fluorescent nanoprobes with MRI, CT, or photoacoustic imaging to complement optical signals. Advances in artificial intelligence and computational image reconstruction can further enhance depth penetration, signal quantification, and real-time monitoring, paving the way for more effective clinical implementation [139]. In summary, biocompatibility, long-term safety, reproducible synthesis, and imaging depth constraints remain key challenges in translating fluorescent nanoprobes from bench to bedside. Overcoming these limitations will require coordinated efforts in materials engineering, toxicology, imaging technology, and regulatory science. Table 6 outlines the critical barriers hindering the clinical translation of fluorescent nanoprobes. Biocompatibility and long-term safety challenges stem from potential cytotoxicity, heavy metal ion release, and organ accumulation. Reproducibility and stability issues arise from synthesis inconsistencies, leading to unreliable fluorescence performance and limited regulatory approval. Imaging depth and temporal resolution constraints restrict in vivo applicability, despite improvements through NIR and upconversion technologies. Addressing these limitations requires a multidisciplinary approach combining nanomaterial design, imaging technology, biosafety evaluation, and regulatory standardization.

## 8. Future Perspectives

### 8.1. Integration with Artificial Intelligence

The integration of artificial intelligence (AI) with fluorescent nanoprobes represents a transformative trend in biomedical imaging and diagnostics. AI algorithms, including machine learning and deep learning, enable automated image processing, enhanced signal analysis, and pattern recognition, which are essential for handling the complex data generated by high-resolution fluorescence imaging [140]. One major application of AI is signal enhancement and noise reduction. Fluorescent nanoprobes, particularly those used in vivo, often encounter background autofluorescence, tissue scattering, and photobleaching artifacts. AI-assisted image reconstruction can correct for these distortions, improving signal-to-noise ratios and providing clearer visualization of biological structures at single-cell resolution [12,141]. For instance, convolutional neural networks (CNNs) have been applied to upconversion nanoparticle imaging data to accurately distinguish tumor margins from healthy tissue in preclinical models [142]. AI also facilitates predictive diagnostics and decision-making. By analyzing fluorescence intensity, ratiometric signals, and spatial distribution patterns, AI models can predict disease progression, therapy response, or metastatic potential. This enables clinicians to personalize treatment regimens based on real-time nanoprobe imaging data, moving toward precision medicine [143]. Moreover, AI-driven analysis supports high-throughput screening in drug development and biomarker discovery. Fluorescent nanoprobes can simultaneously report multiple cellular parameters, such as pH, ion concentration, enzyme activity, and metabolic status. AI algorithms can process these multidimensional datasets to identify correlations and patterns that are otherwise imperceptible, accelerating translational research [144,145,146]. In summary, AI integration enhances the accuracy, speed, and predictive power of fluorescent nanoprobe-based imaging, bridging the gap between raw experimental data and actionable clinical insights.

### 8.2. Multimodal Imaging and Theranostics

Another promising direction is the development of multimodal imaging platforms and theranostic nanoparticles, which combine diagnostic imaging and therapeutic functionalities in a single nanosystem. While fluorescent nanoprobes provide high sensitivity and molecular specificity, their optical signals can be limited by tissue penetration and resolution. Multimodal approaches integrate complementary imaging modalities such as magnetic resonance imaging (MRI), computed tomography (CT), positron emission tomography (PET), and photoacoustic imaging, offering a comprehensive understanding of biological processes in vivo [147]. For example, quantum dots or upconversion nanoparticles can be conjugated with gadolinium or iron oxide cores to enable simultaneous fluorescence and MRI imaging. This allows high-resolution anatomical imaging through MRI while retaining molecular specificity through fluorescence, which is particularly useful for tumor detection, vascular imaging, and lymph node mapping [148]. Similarly, hybrid nanoparticles combining PET isotopes with fluorescent dyes provide sensitive molecular imaging with precise localization. Theranostic nanoparticles integrate imaging with therapeutic functions such as chemotherapy, photodynamic therapy (PDT), or photothermal therapy (PTT). Fluorescent nanoprobes act as both reporters and activators: for instance, upconversion nanoparticles can absorb NIR light to activate a photosensitizer while simultaneously emitting fluorescence to monitor treatment in real time [149]. Dye-doped silica nanoparticles can deliver chemotherapeutics while reporting intracellular drug release via fluorescence intensity changes, providing feedback on therapeutic efficacy and distribution [150]. The combination of imaging and therapy in a single platform not only enhances treatment precision but also reduces systemic toxicity by ensuring drug delivery only at the target site. Multimodal theranostic strategies are poised to play a central role in personalized medicine, enabling clinicians to monitor therapy outcomes and adjust interventions dynamically.

### 8.3. Emerging Trends in Precision Medicine

Fluorescent nanoprobes are increasingly aligned with the goals of precision medicine, which aims to deliver the right treatment to the right patient at the right time. Future trends focus on highly specific, multifunctional, and responsive nanoprobes capable of monitoring multiple disease markers simultaneously and providing real-time feedback on therapy effectiveness. One emerging area is smart, activatable probes that respond to multiple stimuli, including pH, enzyme activity, redox state, or temperature. These nanoprobes can provide spatially and temporally resolved information about tumor microenvironments, inflammation, or metabolic states, enabling clinicians to tailor therapy based on individual disease profiles [151]. Multiplexed imaging is another trend, where nanoparticles encode multiple fluorescence signals to simultaneously monitor several biomarkers or cellular pathways. This approach allows a comprehensive assessment of complex diseases such as cancer or neurodegeneration, where multiple molecular factors influence disease progression [152]. Additionally, integration with wearable and implantable devices is being explored. Fluorescent nanoprobes embedded in bioresponsive hydrogels or microneedles can continuously monitor biomarkers in interstitial fluid, providing real-time feedback for chronic disease management [153]. Finally, the combination of AI, multimodal imaging, and smart probes is likely to transform clinical workflows. These technologies can enable automated, non-invasive diagnostics, longitudinal patient monitoring, and personalized therapy optimization. As the field advances, fluorescent nanoprobes are expected to play a pivotal role in next-generation precision medicine, bridging the gap between molecular imaging and individualized patient care. Table 4 summarizes the emerging directions in fluorescent nanoprobe research that are shaping the future of biomedical imaging and precision medicine. The integration of artificial intelligence enables automated image analysis, noise reduction, and predictive diagnostics, significantly improving the interpretability of complex fluorescence datasets. Multimodal imaging platforms merge the high sensitivity of optical imaging with the anatomical and functional depth of MRI, CT, PET, and photoacoustic modalities, while theranostic nanoparticles unite diagnostic and therapeutic capabilities for image-guided therapy. Additionally, smart and multiplexed nanoprobes offer real-time, multi-stimuli-responsive imaging for dynamic disease monitoring. Finally, the convergence of AI, wearable biosensing, and nanotheranostics is steering the field toward personalized, data-driven healthcare solutions, marking a pivotal advancement in translational nanomedicine. Table 7 provides the emerging directions in fluorescent nanoprobe research that are shaping the future of biomedical imaging and precision medicine. The integration of artificial intelligence enables automated image analysis, noise reduction, and predictive diagnostics, significantly improving the interpretability of complex fluorescence datasets. Multimodal imaging platforms merge the high sensitivity of optical imaging with the anatomical and functional depth of MRI, CT, PET, and photoacoustic modalities, while theranostic nanoparticles unite diagnostic and therapeutic capabilities for image-guided therapy. Additionally, smart and multiplexed nanoprobes offer real-time, multi-stimuli-responsive imaging for dynamic disease monitoring. Finally, the convergence of AI, wearable biosensing, and nanotheranostics is steering the field toward personalized, data-driven healthcare solutions, marking a pivotal advancement in translational nanomedicine.

## 9. Conclusions

Fluorescent nanoprobes have become important tools in biomedical imaging and diagnostics, enabling molecular-level visualization in complex biological systems. Their impact arises not simply from general optical advantages, but from material-specific engineering strategies that optimize quantum efficiency, lifetime behavior, and microenvironmental responsiveness. Advances in nanoparticle design including quantum dots, carbon dots, upconversion nanoparticles, and dye-doped silica platforms have enhanced real-time visualization of biological processes both in vitro and in vivo. A major benefit of these nanoprobes is their capacity for targeted and functional imaging. Surface modifications, bioconjugation approaches, and activatable probe designs allow selective binding to cells, tissues, or molecular markers while reducing off-target interactions. Mechanistic features such as enzyme-triggered activation, redox-responsive fluorescence, and dual-emission ratiometric reporting now provide quantitative insight into dynamic biological pathways, aiding early diagnosis, surgical guidance, and therapeutic monitoring. Despite rapid progress, limitations remain. Challenges include ensuring biocompatibility, minimizing long-term accumulation, achieving reproducible large-scale synthesis, and improving imaging depth. Regulatory and safety considerations also require rigorous evaluation before clinical translation. Current research is addressing these barriers through biodegradable nanomaterials, renally clearable architectures, zwitterionic surface coatings, and precision-controlled synthesis methods. Looking ahead, integration with emerging technologies will further extend the capabilities of fluorescent nanoprobes. Artificial intelligence can enhance image analysis, automate diagnostic interpretation, and support patient-specific predictions. Multimodal imaging platforms that combine fluorescence with MRI, CT, PET, or photoacoustic imaging can overcome depth limitations and provide complementary anatomical and functional data. The development of multiplexed, microenvironment-responsive, and theranostic nanoprobes aligns closely with the goals of precision medicine, enabling simultaneous monitoring of multiple biomarkers and real-time assessment of therapeutic efficacy. In conclusion, fluorescent nanoprobes represent a versatile platform for advancing non-invasive biomedical imaging and disease diagnosis. Continued innovation in optical engineering, surface chemistry, and biodegradability is expected to significantly enhance their clinical utility, supporting earlier detection, more accurate intervention, and real-time monitoring in next-generation precision healthcare.

## Figures and Tables

**Table 1:** Fundamental principles, key properties, and comparative advantages of fluorescent nanoprobes.

Aspect/Category	Core Concept and Mechanism	Biomedical Significance/Impact	Representative Examples/Key Features	References
**Basic Principles of Fluorescence**	Fluorescence arises from photon absorption, excitation to a higher energy state, and emission at a longer wavelength (Stokes shift). Core parameters include quantum yield and fluorescence lifetime.	Determines brightness, sensitivity, and contrast in biomedical imaging; essential for rational probe design.	Quantum dots and carbon dots exhibit high quantum yields; lifetime imaging improves precision compared with intensity-based detection.	[15,16,17,18]
**Fluorescence Lifetime Imaging and Lifetime-Based Environmental Sensing (Unified Entry)**	Measures decay time instead of intensity. Lifetime responds to microenvironmental factors such as pH, viscosity, oxygen level, and molecular interactions.	Provides concentration-independent contrast, reduces autofluorescence interference, and enables real-time sensing of metabolic or biochemical changes.	Used to map enzyme activity, metabolic states, drug-release dynamics, and intracellular environment variations in living tissues.	[17,18,24,25,26]
**Optical Penetration and NIR Imaging**	NIR and NIR-II wavelengths penetrate deeper into tissues due to lower scattering and absorption.	Enables high-resolution, deep-tissue visualization suitable for in vivo imaging.	NIR-II quantum dots, carbon dots, and upconversion nanoparticles visualize tumors and vasculature several millimeters below the surface.	[12,19]
**Activatable and Responsive Fluorescent Nanoprobes**	Probes switch from “off” to “on” in response to biochemical triggers (enzymes, pH, redox).	Enhances site-specific imaging and reduces background noise.	Enzyme-activated carbon dots; redox-responsive silica nanoprobes for tumor and inflammation detection.	[20]
**Key Optical Parameters**	Includes absorption/emission spectra, brightness, quantum yield, photostability, and lifetime.	Influences imaging sensitivity, durability under illumination, and quantitative accuracy.	Quantum dots and UCNPs offer high photostability; NIR-II probes reduce tissue autofluorescence.	[19,21,22,23]
**Physicochemical Properties (Size, Surface, Stability)**	Size, charge, and surface chemistry regulate colloidal stability, biodistribution, and clearance routes.	Ensures predictable in vivo behavior and contributes to safety.	Nanoparticles < 6 nm undergo renal clearance; PEGylated or zwitterionic coatings reduce protein adsorption.	[27,28]
**Degradability and Biocompatibility**	Biodegradable and carbon-based nanoprobes break down into non-toxic products.	Reduces long-term accumulation and improves clinical applicability.	Carbon dots and organic nanoprobes degrade naturally, minimizing organ retention.	[29,30,31]
**Brightness and Sensitivity**	Multiple fluorophores or quantum confinement effects amplify emission.	Enables imaging at low probe concentrations, reducing systemic exposure.	Quantum dots and dye-doped silica nanoparticles enable bright, sensitive detection at cellular resolution.	[32]
**Photostability**	Resistance to photobleaching maintains stable emission during prolonged imaging.	Essential for intraoperative visualization and long-term monitoring.	Quantum dots, UCNPs, and metal nanoclusters sustain emission under continuous excitation.	[33]
**Spectral Tunability**	Emission wavelength controlled by nanoparticle size, dopant type, or surface chemistry.	Supports multiplex imaging to track multiple biological targets simultaneously.	Size-tuned quantum dots and doped UCNPs covering visible to NIR spectra.	[34]
**Activatable and Ratiometric Designs**	Dual- or multi-emission systems enable internal calibration or environment-triggered signal changes.	Improves measurement accuracy for dynamic biological processes.	pH-sensitive and redox-responsive carbon dots for ratiometric biosensing.	[35]
**Multifunctionality and Theranostics**	Combines imaging and therapy on a single nanoprobe (drug loading, photothermal agents).	Enables real-time tracking of treatment response and image-guided therapy.	Quantum dots or silica nanoprobes integrated with drugs and targeting ligands.	[36]
**Deep-Tissue and Intraoperative Imaging**	NIR-II nanoprobes achieve enhanced contrast and spatial resolution beyond visible-range dyes.	Supports early detection and clear visualization of tumors and vasculature during surgery.	NIR-II emitting nanoprobes for cancer diagnosis and surgical navigation.	[37]

**Table 2:** Comparative summary of major classes of fluorescent nanoprobes: structural design, optical behavior, and biomedical applications.

Class of Nanoprobe	Core Structure and Mechanism	Key Optical and Physicochemical Properties	Biomedical Applications and Functional Advantages	Representative References
**Quantum Dots (QDs)**	Semiconductor nanocrystals (2–10 nm) composed of a core (CdSe, CdTe, InP) and a passivating shell (ZnS, ZnSe) that enhances quantum yield and stability. Optical emission arises from quantum confinement, where energy levels are size-dependent.	• Size-tunable emission across visible to NIR spectrum • Broad absorption and narrow, symmetric emission peaks • High quantum yield and exceptional photostability • Surface modifiable with thiols, phosphines, or polymers for aqueous stability • Potential cytotoxicity due to heavy metals	• **Cancer Imaging:** HER2-targeted QDs for visualization of breast tumors and micrometastases • **Vascular Imaging:** Long-circulating QDs enable high-resolution angiography • **Neurological Studies:** Synaptic vesicle protein–conjugated QDs track neuronal dynamics • **Infectious Disease Diagnostics:** QD-based immunoassays detect viral proteins at femtomolar levels • **Intraoperative Guidance:** NIR-emitting QDs delineate tumor margins and sentinel lymph nodes • **Advancements:** Development of Cd-free QDs (InP/ZnS, carbon QDs) to reduce toxicity	[12,30,38,39,40,41,42,43,44,45,46,47,48,49]
**Carbon Dots (CDs)**	Nanoscale carbon particles (<10 nm) with graphitic or amorphous carbon cores and abundant surface functional groups (–COOH, –OH, –NH_2_). Synthesized via bottom-up (pyrolysis, hydrothermal, microwave) or top-down (laser ablation, oxidation) methods.	• Excellent **photostability** and **biocompatibility** • Tunable fluorescence from visible to NIR via heteroatom doping (N, S, P) • Excitation-dependent emission and long fluorescence lifetime • Small size allows renal clearance • Environment-sensitive luminescence (pH, ions, ROS)	• **Cancer Imaging:** Folic acid– or transferrin-functionalized CDs selectively target tumor cells • **Biosensing:** Detection of metal ions (Cu^2+^, Hg^2+^, Fe^3+^), biomolecules (glucose, dopamine), and ROS • **Neuroimaging:** Dopamine-sensitive CDs monitor neurotransmitter dynamics; cross the BBB • **NIR Imaging:** NIR-emitting CDs visualize vascular structures and tumor angiogenesis • **Theranostics:** Doxorubicin-loaded CDs for image-guided chemotherapy • **Photothermal/PDT:** Light-activated CDs for cancer cell ablation • **Tissue Engineering:** Incorporated into hydrogels and scaffolds for imaging and cell growth	[11,14,31,43,48,50,51,52,53,54,55,56,57,,58,59,60,61,62,63,64,65,66,67]
**Upconversion Nanoparticles (UCNPs)**	Inorganic crystalline hosts (e.g., NaYF_4_) doped with lanthanide ions (Yb^3+^, Er^3+^, Tm^3+^). Emit higher-energy photons upon sequential absorption of multiple NIR photons via energy transfer upconversion (ETU), excited-state absorption (ESA), or photon avalanche (PA).	• Anti-Stokes emission (NIR → visible/UV) • Sharp, narrow emission bands and long lifetimes • High photostability and low autofluorescence • Deep tissue penetration via NIR excitation • Enhanced quantum yield with core–shell architectures • Resistant to photobleaching and scattering artifacts	• **Deep-Tissue Imaging:** Enables visualization several millimeters beneath the surface due to minimal tissue absorption • **Cancer Detection:** Antibody-conjugated UCNPs identify tumor biomarkers • **Intraoperative Navigation:** Real-time fluorescence for tumor resection • **Theranostics:** UCNPs activate photosensitizers for PDT and deliver drugs • **Multiplex Imaging:** Different dopant ions yield multiple emission colors under one excitation • **High S/B Ratio:** Reduced background autofluorescence in biological tissues	[18,56,68,69,70,71,72,73,74,75,76,77,78,79,80]
**Dye-Doped Silica Nanoparticles (DDSNs)**	Organic fluorescent dyes encapsulated or covalently bound within an amorphous silica matrix formed via sol–gel or microemulsion synthesis. The silica shell prevents dye leaching and aggregation-induced quenching.	• High chemical and photostability • Tunable porosity and thickness • Minimal dye aggregation and sustained brightness • Easily functionalized surface (PEG, antibodies, aptamers) • Biocompatible and non-toxic • Supports multiplexed or ratiometric fluorescence	• **Cancer Imaging:** Tumor-targeted DDSNs visualize margins and detect circulating tumor cells • **Biosensing:** Encapsulated pH- and ion-sensitive dyes for intracellular monitoring • **Pathogen Detection:** Antibody-functionalized DDSNs for bacteria/virus assays • **Image-Guided Drug Delivery:** Mesoporous DDSNs co-loaded with chemotherapeutics and dyes for simultaneous therapy tracking • **Multiplex Biomarker Detection:** Multi-dye DDSNs for multi-analyte diagnostics • **Hybrid Platforms:** Integration with magnetic or plasmonic materials for multimodal imaging	[26,81,82,83,84,85,86,87,88,89,90,91]

**Table 3:** Comparative summary of functionalization and targeting strategies for fluorescent nanoprobes.

Strategy/Probe Type	Key Mechanism or Functional Approach	Advantages and Biomedical Impact	Representative Examples/Applications	References
**Surface Modification and Bioconjugation**	Surface coatings (PEG, Zwitterionic polymers, Dextran, chitosan); Covalent (Carbodiimide, Click chemistry) and noncovalent (Electrostatic, hydrophobic, Biotin–streptavidin) conjugation	Enhances colloidal stability, Solubility, and Circulation time, and reduces nonspecific interactions; Enables specific ligand attachment for biosensing or targeting	PEGylated quantum dots for prolonged circulation; Silica-coated UCNPs for reduced toxicity; Amino-functionalized carbon dots for biocompatibility	[92,93,94,95,96]
**Tumor-Targeted and Organ-Specific Probes**	Passive targeting via EPR effect; active targeting using ligands such as antibodies, Peptides, aptamers, or Small molecules	Enables selective accumulation in tumor or organ-specific tissues; Improves imaging contrast and reduces systemic toxicity	Trastuzumab-modified QDs for HER2 imaging; Folic acid-conjugated CDs for ovarian cancer; angiopep-2 peptides for brain targeting; glycyrrhetinic acid-modified NPs for liver imaging	[97,98,99,100]
**Activatable Probes**	Designed to respond to physiological or pathological stimuli such as low pH, GSH concentration, Hypoxia, or enzyme activity	Provides “off–on” fluorescence activation, improving specificity and minimizing background signal	pH-responsive UCNPs for acidic tumor detection; Enzyme-cleavable peptide–carbon dots for protease activity imaging	[101,102]
**Ratiometric Probes**	Utilize two emission bands: one responsive to analyte changes and one constant as an internal reference	Allow quantitative imaging independent of probe concentration, photobleaching, or uneven illumination	Dual-emission CDs for intracellular pH sensing; dual-dye DDSNs for ion detection; UCNP-based ratiometric oxygen sensors for tumor hypoxia imaging	[103,104]

**Table 4:** Comparative overview of biomedical applications of fluorescent nanoprobes.

Application Area	Mechanism or Working Principle	Biomedical Advantages and Impact	Representative Examples/Probe Types	References
**Early Disease Detection and Diagnosis**	Targeted fluorescence of biomarkers via ligand–nanoprobe conjugation; detection of disease-associated biomolecules at low concentrations	Enables ultra-sensitive and early identification of cancer, infection, and metabolic disorders; supports preclinical and clinical diagnostics	Antibody-conjugated QDs for tumor antigen detection; folic acid–functionalized CDs for cancer imaging; UCNP-based immunoassays for viral antigen detection	[91,105,106,107,108]
**Image-Guided Surgery**	Real-time intraoperative fluorescence visualization of diseased tissue; NIR or visible light imaging for tumor margin delineation	Enhances surgical precision and reduces recurrence; provides high-contrast imaging and deep-tissue visualization	NIR QDs and UCNPs for deep-tissue imaging; targeted CDs and DDSNs for tumor margin detection; activatable probes for enzyme-triggered fluorescence	[48,109,110]
**Real-Time Monitoring of Therapeutic Response**	Fluorescent signal variation with therapeutic activity (e.g., drug release, apoptosis, ROS generation)	Enables real-time assessment of therapy efficacy and treatment adjustment; supports personalized medicine	QDs, CDs, and DDSNs as theranostic platforms for drug delivery and tracking; UCNPs for photodynamic therapy monitoring; activatable and ratiometric probes for oxidative stress imaging	[111,112,113,114]
**In Vivo Biosensing and Tracking**	Dynamic tracking of labeled cells, molecules, or ions within living organisms; fluorescence-based biosensing of physiological changes	Allows non-invasive monitoring of cell migration, immune responses, and metabolic processes in vivo	QDs or CDs for immune cell tracking; UCNPs for deep-tissue biosensing; DDSNs for pH, glucose, and ROS detection in live models	[48]

**Table 5:** Key stages, challenges, and illustrative examples in clinical translation of fluorescent nanoprobes.

Stage/Aspect	Critical Focus/Barrier	Mitigation Strategies/Considerations	Illustrative Examples or Trends	References
**Preclinical Characterization & Safety**	Ensuring reproducible nanoprobe physicochemical properties (size, charge, fluorescence), assessing cytotoxicity, stability, and cellular uptake	Rigorous standardization, long-term in vitro assays, surface passivation and coatings to suppress ion leaching	Use of cadmium-free QDs, PEG or lipid coatings to improve biocompatibility	[117,118,119,120,121]
**In Vivo Biodistribution & Pharmacokinetics**	Achieving favorable tumor accumulation while minimizing off-target organ retention and long-term persistence	Optimization of size (<10 nm for renal clearance), active targeting ligands, stealth coatings, real-time imaging of distribution	Studies exploring UCNPs or silica-coated probes in rodent tumor models	[119]; also see clinical-translation reviews
**Toxicity and Biosafety Evaluation**	Avoiding dose-dependent toxicity, immunogenicity, complement activation, accumulation in reticuloendothelial organs	Employ inert matrix materials (silica, carbon dots), surface shielding (PEG, zwitterions), detailed long-term toxicity studies	Transition to silicon QDs, biocompatible nanoprobes such as dye-doped silica or carbon dots	[119,120]
**Regulatory & Manufacturing Challenges**	Meeting standards for reproducibility, batch-to-batch consistency, quality control, and regulatory classification (diagnostic agent, device, or combination product)	Early engagement with regulatory bodies, adherence to GMP-like practices, simplified nanoprobe designs	Pilot translation efforts in sentinel lymph node imaging using silica-encapsulated dyes or ICG-loaded nanoparticles	[122,123,124]
**Clinical Feasibility and Trials**	Demonstrating safety, biodistribution, diagnostic/therapeutic efficacy, and patient benefit in early-phase trials	Well-designed Phase I/II studies, careful dose escalation, imaging endpoints, comparison with standard-of-care diagnostics	Clinical trials with ICG-silica nanoparticles for sentinel lymph node mapping, and cadmium-free QDs in pilot imaging studies	[124,125]
**Translational Trends & Future Directions**	Integration with multimodal imaging, AI-assisted image analysis, simplified architectures to reduce complexity, regulatory harmonization	Combine fluorescence with MRI, PET, or photoacoustic modalities; use smart or activatable probes; standardize translational pathways	Review emphasis on NIR-II probes under evaluation, and challenges in scaling targeted fluorescent probes for intraoperative navigation	

**Table 6:** Major challenges and limitations in the clinical translation of fluorescent nanoprobes.

Challenge Area	Underlying Cause/Mechanism	Impact on Biomedical Application	Proposed Mitigation Strategies	References
**Biocompatibility and Long-Term Safety**	Interaction of nanoparticles with biological systems influenced by size, charge, and surface chemistry; release of toxic ions (e.g., Cd^2+^, Pb^2+^) from semiconductor QDs; accumulation of non-biodegradable materials in organs	Potential cytotoxicity, oxidative stress, inflammation, immune activation, and long-term organ retention hinder clinical use	Use of cadmium-free or silicon-based QDs; application of biocompatible materials (carbon dots, DDSNs, UCNPs); PEGylation, zwitterionic or lipid coatings to improve circulation and reduce immune response	[127,128,129,130]
**Stability and Reproducibility of Synthesis**	Variations in precursor purity, synthesis temperature, dopant ratios, and reaction time lead to inconsistent particle size, morphology, or optical properties	Batch-to-batch variability causes inconsistent fluorescence output, targeting accuracy, and quantitative imaging reliability	Development of standardized and scalable synthesis protocols; quality control during nanocrystal growth; surface passivation; rigorous control of dopant concentration and reaction kinetics	[131,132,133]
**Imaging Depth and Real-Time Monitoring Constraints**	Light scattering, Absorption, and Autofluorescence in tissues limit penetration depth; Dynamic physiological processes affect temporal signal stability	Restricts visualization of deep-seated or moving tissues; Reduces imaging resolution and accuracy in vivo	Employ near-infrared (NIR-I/NIR-II) or upconversion nanoprobes; integrate multimodal imaging (MRI, CT, photoacoustic); use AI-assisted image reconstruction and advanced optical detectors	[104,134,135,136,137]

**Table 7:** Future perspectives and emerging innovations in fluorescent nanoprobe research.

Emerging Direction/Technology	Key Principles and Mechanisms	Advantages and Biomedical Implications	Representative Examples/Applications	References
**Integration with Artificial Intelligence (AI)**	Application of machine learning (ML) and deep learning (DL) algorithms for image analysis, signal processing, and pattern recognition of fluorescence imaging data	Enhances imaging accuracy, reduces background noise, and enables automated quantitative analysis; facilitates real-time diagnostic decision-making and predictive modeling	CNN-assisted identification of tumor margins using UCNP images; AI-based signal reconstruction for background autofluorescence correction; predictive algorithms for therapy response and disease progression	[138,139,140,141]
**AI for Data-driven Biomarker Discovery and Drug Screening**	High-throughput AI algorithms analyzing large fluorescence datasets from multiplexed probes for biomarker identification	Accelerates identification of molecular signatures and drug responses; enables multidimensional correlation analysis of optical, biochemical, and morphological data	AI processing of multiplexed carbon dot or QD fluorescence signals to identify novel biomarkers for early cancer and inflammatory disease detection	[142,143,144]
**Multimodal Imaging Systems**	Integration of fluorescence imaging with complementary modalities (MRI, CT, PET, photoacoustic imaging) within a single nanoparticle platform	Provides synergistic anatomical and molecular information; improves spatial resolution, imaging depth, and diagnostic accuracy	QDs or UCNPs conjugated with gadolinium or Fe_3_O_4_ for MRI-fluorescence dual imaging; PET-fluorescence hybrids for precise tumor localization	[145,146]
**Theranostic Nanoparticles**	Combination of therapeutic (drug delivery, PDT/PTT) and diagnostic (fluorescence imaging) functions in a single nanoprobe	Enables image-guided therapy, real-time tracking of treatment efficacy, and reduced off-target toxicity	UCNPs activating photosensitizers under NIR light for PDT; dye-doped silica nanoparticles co-loaded with chemotherapeutics for image-guided drug release	[147,148]
**Smart and Activatable Nanoprobes**	Fluorescent probes responsive to multiple physiological stimuli such as pH, enzyme activity, redox potential, and temperature	Provides spatiotemporal imaging of dynamic biological processes and tumor microenvironments; enhances diagnostic specificity	Dual-responsive probes that emit distinct signals under acidic and enzymatic conditions in cancer tissues; ROS-sensitive carbon dots for inflammation tracking	[149]
**Multiplexed and Multicolor Imaging**	Encoding multiple fluorophores within one nanoprobe or using spectral separation to monitor several biomarkers simultaneously	Enables simultaneous tracking of multiple cellular pathways or disease markers; enhances diagnostic depth in complex diseases	Multicolor QDs or CDs enabling simultaneous imaging of cancer biomarkers, neurotransmitters, and inflammatory mediators	[150]
**Integration with Wearable/Implantable Devices**	Embedding nanoprobes in microneedles, bioresponsive hydrogels, or flexible implants for continuous optical biosensing	Allows real-time, minimally invasive monitoring of metabolites and biomarkers in interstitial fluids or tissues	Fluorescent nanoprobe-integrated microneedles for glucose or lactate sensing; hydrogel-based sensors for pH and ion fluctuations in chronic diseases	[151]
**Convergence toward Precision Medicine**	Synergistic integration of AI, multimodal imaging, and smart nanoprobes for individualized diagnosis and treatment	Enables non-invasive, longitudinal patient monitoring and adaptive therapy optimization; supports precision oncology and chronic disease management	AI-guided multimodal theranostic platforms using UCNPs or dye-doped silica nanoprobes for personalized image-guided interventions	[138]

## Data Availability

No new data were created or analyzed in this study.
